# ToxR is a c-di-GMP binding protein that modulates surface-associated behaviour in *Pseudomonas aeruginosa*

**DOI:** 10.1038/s41522-022-00325-9

**Published:** 2022-08-18

**Authors:** Jean-Frédéric Dubern, Manuel Romero, Anne Mai-Prochnow, Marco Messina, Eleftheria Trampari, Hardeep Naghra-van Gijzel, Kok-Gan Chan, Alessandro M. Carabelli, Nicolas Barraud, James Lazenby, Ye Chen, Shaun Robertson, Jacob G. Malone, Paul Williams, Stephan Heeb, Miguel Cámara

**Affiliations:** 1 National Biofilms Innovation Centre, Biodiscovery Institute and School of Life Sciences, University of Nottingham, Nottingham, UK; 2 Department of Molecular Microbiology, John Innes Centre, Norwich, UK; 3 Institute of Biological Sciences, Faculty of Science, University of Malaya, Kuala Lumpur, Malaysia; 4 International Genome Centre, Jiangsu University, Zhenjiang, China; 5 School of Pharmacy, Boots Science Building, University of Nottingham, Nottingham, UK; 6 Centre for Marine Bio-Innovation, School of Biotechnology and Biomolecular Science, University of New South Wales, Sydney, Australia; 7 School of Biological Sciences, University of East Anglia, Norwich, UK; 8 Present Address: School of Chemical and Biomolecular Engineering, University of Sydney, Sydney, Australia; 9 Present Address: Department of Science, University Roma Tre, Rome, Italy; 10 Present Address: Quadram Institute Bioscience, Norwich Research Park, Norwich, UK; 11 Present Address: Genomic Sciences, GlaxoSmithKline Research and Development, Stevenage, UK; 12 Present Address: Department of Medicine, University of Cambridge, Cambridge, UK; 13 Present Address: Genetics of Biofilms Unit, Institut Pasteur, Paris, France; 14 Present Address: Q Squared Solutions, Crystal Plaza, Pudong, Shanghai, China

**Keywords:** Biofilms, Microbial genetics

## Abstract

*Pseudomonas aeruginosa* uses multiple protein regulators that work in tandem to control the production of a wide range of virulence factors and facilitate rapid adaptation to diverse environmental conditions. In this opportunistic pathogen, ToxR was known to positively regulate the production of the major virulence factor exotoxin A and now, through analysis of genetic changes between two sublines of *P. aeruginosa* PAO1 and functional complementation of swarming, we have identified a previously unknown role of ToxR in surface-associated motility in *P. aeruginosa*. Further analysis revealed that ToxR had an impact on swarming motility by regulating the Rhl quorum sensing system and subsequent production of rhamnolipid surfactants. Additionally, ToxR was found to tightly bind cyclic diguanylate (c-di-GMP) and negatively affect traits controlled by this second messenger including reducing biofilm formation and the expression of Psl and Pel exopolysaccharides, necessary for attachment and sessile communities matrix scaffolding, in *P. aeruginosa*. Moreover, a link between the post-transcriptional regulator RsmA and *toxR* expression via the alternative sigma factor PvdS, induced under iron-limiting conditions, is established. This study reveals the importance of ToxR in a sophisticated regulation of free-living and biofilm-associated lifestyles, appropriate for establishing acute or chronic *P. aeruginosa* infections.

## Introduction

*Pseudomonas aeruginosa* is an opportunistic pathogen able to adopt different free-living or biofilm-associated lifestyles, appropriate for establishing acute or chronic infections. This bacterium produces a wide range of virulence-associated factors including proteases, pyocyanin, exotoxin A, rhamnolipids and hydrogen cyanide^1^ in a population-dependent manner through quorum sensing (QS)-mediated mechanisms^2^. These include two *N*-acylhomoserine lactone (AHL) signal-dependent QS systems, the RhlR-RhlI and LasR-LasI systems. LasI synthesizes *N*-(3-oxododecanoyl)-l-homoserine lactone (3-oxo-C12-HSL) and RhlI, *N*-butanoyl-l-homoserine lactone (C4-HSL) which activate their cognate regulators, LasR and RhlR respectively, leading to the regulation of multiple virulence traits^2^.

In addition to QS, *P. aeruginosa* uses post-transcriptional regulators to facilitate rapid adaptation to diverse environmental conditions and regulate virulence^3^. One of these, the RNA binding protein RsmA, has a significant impact on ~9% of the gene transcripts from *P. aeruginosa* in both a negative and positive fashion^4^. This small RNA-binding protein belongs to the CsrA family of post-transcriptional regulators, initially described in *Escherichia coli*^5^, that interacts with ANGGA motifs situated within RNA hairpins^6,7^. As a result, RsmA represses the translation of genes involved in the establishment of chronic biofilm infections, including transcripts from genes coding for type VI secretion systems and exopolysaccharide (EPS) biosynthesis^8,9^. Additionally, RsmA positively influences phenotypes related to acute infections such as motility and type III secretion systems^10,11^.

Moreover, RsmA regulates lifestyles in *P. aeruginosa* via controlling the intracellular levels of cyclic diguanylate (c-di-GMP)^12^, a global modulator of complex cellular processes at different levels: transcriptional, post-transcriptional and post-translational^13^. C-di-GMP is synthesized by diguanylate cyclases (DGCs) and hydrolysed by phosphodiesterases (PDEs)^14–17^. Proteins containing a GGDEF domain (responsible for DGC activity), an EAL domain and/or an HD-GYP motif (both responsible for PDE activity) have been identified in a large number of bacterial genomes^16,18–20^. In particular, it is accepted that c-di-GMP levels control phenotypic changes related to the transition between motility and sessility, with high levels promoting biofilm formation and low levels leading to biofilm detachment and increased motility such as swarming^15,21,–23^.

Swarming is a characteristic mode of surface translocation used by a wide range of bacteria^24^. In *P. aeruginosa* this motility occurs on semisolid surfaces, for example low percentage agar plates, where this bacterium can form a distinctive pattern of radiating tendrils^21^. *P. aeruginosa* swarming is mediated by both flagellum and type IV pili, and requires rhamnolipids and QS activity as it is the result of a collective behaviour^25–27^. Rhamnolipids are believed to support migration initiation or seeding dispersal^28^ by lowering surface tension and thus allowing flagella-based propulsion of *P. aeruginosa* cells^29^. Rhamnolipid production is upregulated under iron-limited conditions and correlates with the formation of flat, unstructured biofilms^30^. It involves the activity of several enzymes coded by the *rhl* genes, including the acyltransferase activity of RhlA, the rhamnosyl transferase activities of RhlB and RhlC to form mono- and di-rhamnolipids respectively^31–34^. Expression of *rhlA*, *rhlB* and *rhlC* have all been shown to be under the control of the QS transcription factor RhlR^31,32,34,35^.

The major virulence factor exotoxin A (ETA, encoded by *toxA*), originally described by Liu et al.^36,37^, is a highly specific ADP-ribosyltransferase released by *P. aeruginosa*^38^. In the context of infection, ETA exerts its enzymatic activity within the target host cell by specific ADP-ribosylation of the highly conserved diphthamide residue in the eukaryotic elongation factor-2^39^. As a result, protein biosynthesis is hindered leading to cell cycle arrest and apoptosis^40^. The regulation of *toxA* expression is complex and several studies have established a relation between ETA production and iron metabolism^41–43^. A regulatory element influencing ETA production was first discovered by the research group of Galloway^44^ and later designated *toxR*, for toxin regulation^45^. ToxR was found to positively regulate ETA production since overexpressing *toxR* in trans increased ETA yields 10-fold^44^.

In this study we sought to identify genetic elements responsible for differences in the swarming motility of two sublines of *P. aeruginosa* PAO1: PAO1-Nottingham (PAO1-N) and PAO1-Lausanne (PAO1-L). Genome sequence data revealed fundamental differences between these two sublines, including a 59-kb deletion containing *toxR* and a mutation in the c-di-GMP-degrading PDE BifA in PAO1-N. Among the genes from PAO1-L able to restore swarming deficiency to a PAO1-N Δ*rsmA* mutant, *toxR* was found to impact on swarming through regulation of the *rhl* QS system and subsequent rhamnolipid production. Interestingly, ToxR was found to bind c-di-GMP tightly and to negatively affect EPS production and biofilm formation in *P. aeruginosa*. Moreover, a link between RsmA regulation and *toxR* expression via the alternative sigma factor PvdS is established. Here we propose a model for the regulation of surface-associated behaviour via RsmA, PvdS and ToxR and demonstrate the previously unknown involvement of ToxR in the c-di-GMP regulated phenotypes of swarming and biofilm formation.

## Results

### Genetic elements from *P. aeruginosa* PAO1-Lausanne subline restore swarming deficiency in PAO1-Nottingham Δ*rsmA*

**Fig. 1:**
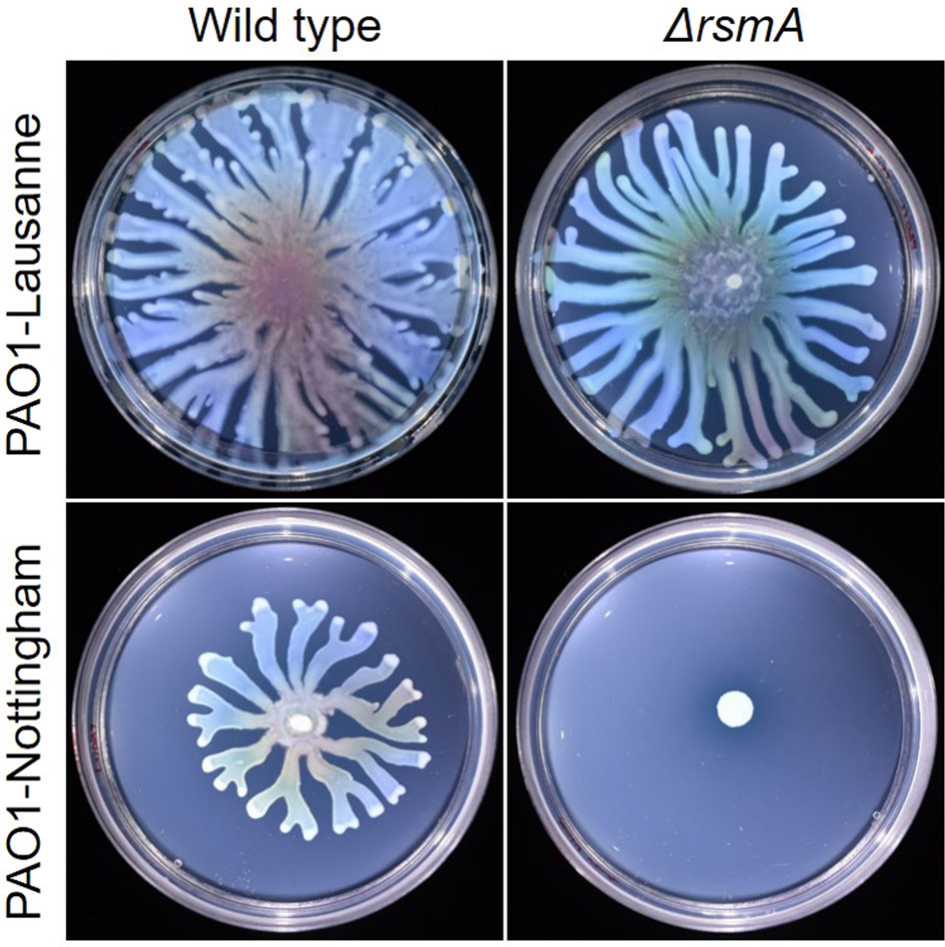
Impact on swarming of an *rsmA* mutation in PAO1-L and PAO1-N. Representative images of swarming motilities displayed by *P. aeruginosa* PAO1-L and PAO1-N WT strains and their *rsmA* mutant derivatives. Swarming plates were incubated at 37 °C for 16 h.

While investigating surface-associated motility in *rsmA* mutants of *P. aeruginosa* PAO1, clear differences in swarming motility between sublines originating from different laboratories were observed. Consistent with previous studies, swarming was abolished entirely in a Δ*rsmA* mutant made in the PAO1-N subline from Nottingham^46^, whereas the same deletion made in the PAO1-L subline from Lausanne had only a modest effect on motility (Fig. 1). Moreover, swarming motility was reduced in the wild type (WT) background of PAO1-N with respect to PAO1-L WT (Fig. 1), suggesting the existence of distinct genetic element(s) that contribute to this differential behaviour.

To discover component(s) within the PAO1-L genome able to restore swarming in the PAO1-N Δ*rsmA* mutant other than *rsmA* itself, random Sau3AI chromosomal DNA fragments from PAO1-L (2–4 kb) were cloned into pME6000, transformed into the Δ*rsmA* mutant of PAO1-N and the resulting clones (~4,000, 92% genomic coverage) screened for restoration of swarming motility. Thirteen clones partially complemented swarming motility in the PAO1-N Δ*rsmA* mutant (Supplementary Fig. 1), suggesting that their chromosomal DNA inserts incorporated genes absent or downregulated in PAO1-N. To isolate the genetic elements able to complement swarming when overexpressed, ORFs in the selected inserts were individually sub-cloned and seven genes were identified. Amongst these, several genes encoding hypothetical proteins (*PA0285*, *PA2072*, *PA2567*) were found. In addition, *PA3825* and *proE* coding for c-di-GMP-specific phosphodiesterases^47,48^ were isolated. Other genes which restored swarming motility in the PAO1-N Δ*rsmA* mutant were *rsmN* encoding the small RNA-binding protein RsmN^3^, which was present in three of the thirteen clones indicating a good level of coverage of the PAO1-L genome, and unexpectedly *toxR*, a gene coding for the regulator of ETA production ToxR. Interestingly, six of the genes found to restore swarming in PAO1-N Δ*rsmA* mutant showed conserved domains from the family of proteins involved in c-di-GMP metabolism and hence would be expected to have an impact on swarming through the modulation of c-di-GMP levels (Table 1 and Supplementary Fig. 1).

**Table 1:** Genetic elements with conserved domains from the family of proteins involved in c-di-GMP metabolism that restored swarming motility in PAO1-N Δ*rsmA* mutant.

Locus ID	Gene product name	C-di-GMP domain
PA5295	ProE	EAL and GGDEF domains, PDE activity shown^47^
PA2072	Conserved hypothetical protein	EAL and GGDEF domains^48^
PA0285	Conserved hypothetical protein	EAL and GGDEF domains^48^
PA3825	Hypothetical protein	EAL domain^48^
PA2567	Hypothetical protein	EAL and GGDEF domains, PDE activity shown^54^
PA0707	ToxR	EAL domain^66^

**Fig. 2:**
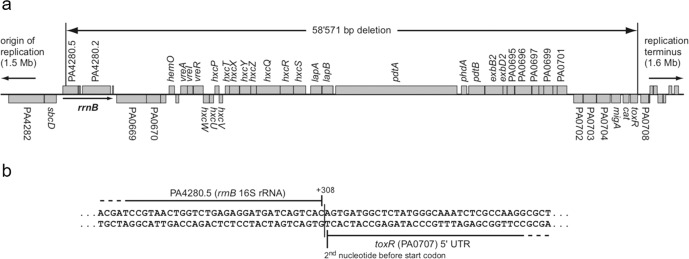
Genetic organization of the 59Kb chromosomal deletion absent from PAO1-N. **a** Genetic organization of the ~59 kb deletion in *P. aeruginosa* PAO1-N subline. **b** The deletion spans from the 308^th^ bp of the 16 S rRNA gene PA4280.5 until the 2nd bp before the start codon of *toxR*.

To determine which of the gene(s) identified was involved in the altered swarming motility of PAO1-N with respect to PAO1-L, the extent of the genetic differences between sublines was investigated using whole-genome sequencing, and the assembled chromosomes of both sublines were compared with the reference genome of PAO1-UW^49^. Candidate single nucleotide polymorphisms (SNPs) as well as small insertions/deletions (INDELs) between sublines were then selected (Supplementary Table 1). Additionally, optical restriction mapping was performed to detect large scale chromosomal rearrangements, deletions and insertions^50,51^. Although none of the previously identified genes was affected by the SNPs identified when comparing genomes from both sublines, sequencing analysis revealed a large deletion (~59 kb) in the PAO1-N genome that included the *toxR* gene (Fig. 2 and Supplementary Table 2). These results strongly suggested that ToxR might play a role in the control of swarming motility. Optical restriction mapping revealed that neither PAO1-N nor PAO1-L sublines have the large 2-Mb *rrnA*/*rrnB* chromosomal inversion reported for PAO1-UW^49^ (Supplementary Fig. 2), and sequencing results indicated that both carry the 12-kb RGP42 island initially found in PAO1-DSM^52^, excluding these as possible differences between the sublines.

### A triple mutation of *rsmA*, *toxR* and *bifA* abolishes swarming in PAO1-L

**Fig. 3:**
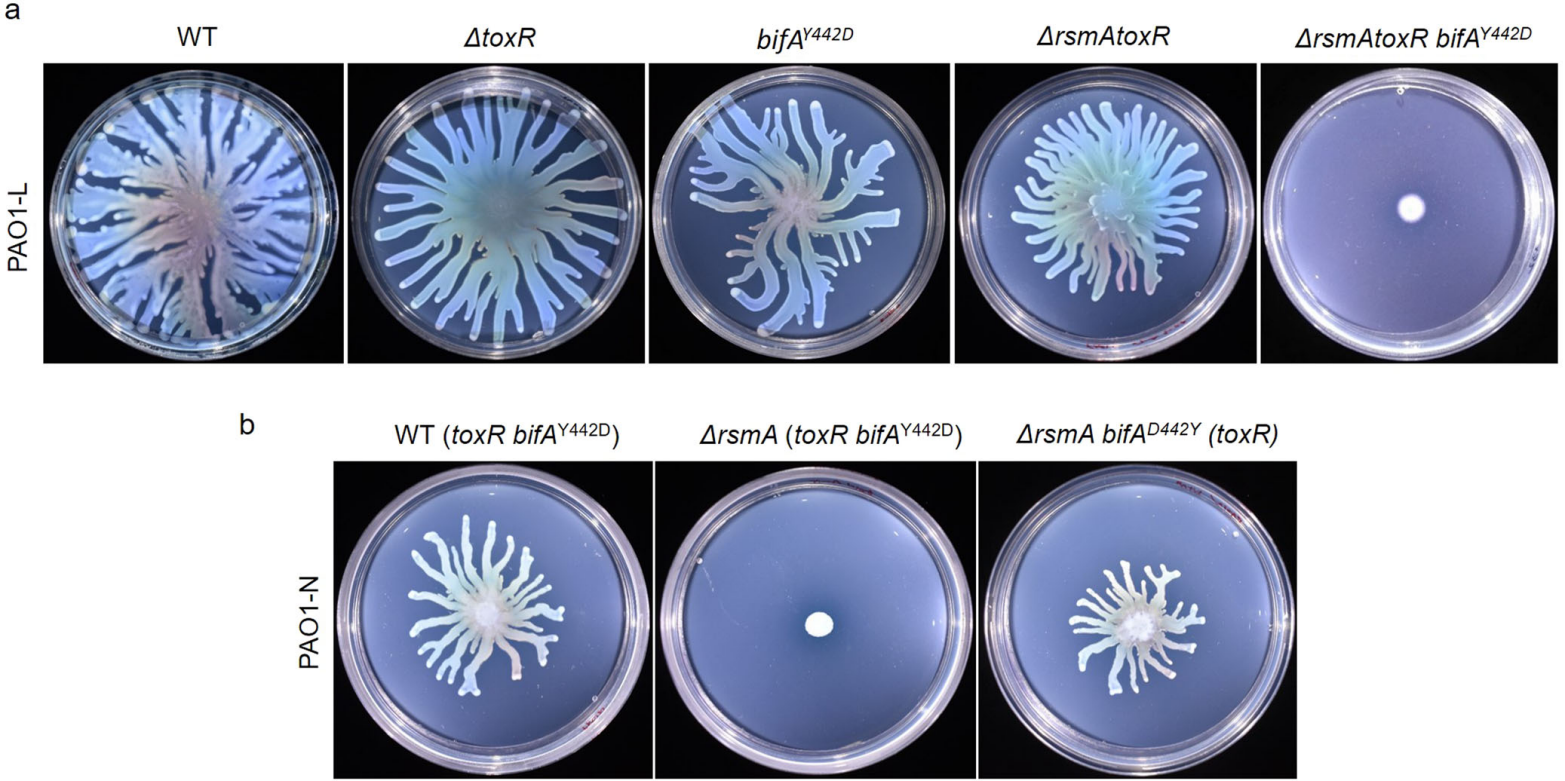
Impact of *rsmA*, *toxR*, *bifA* mutations on swarming. Swarming motilities of PAO1-L **a** and PAO1-N **b** WT strains and their corresponding *rsmA*, *toxR* and *bifA* mutants, assayed over a period of 16 h at 37 °C.

To investigate the role of ToxR in swarming, the impact of *toxR* deletion and the double deletion Δ*rsmA* Δ*toxR* on PAO1-L swarming motility was assessed. Although deletion of *toxR* resulted in a slight reduction in swarming motility, supporting a link with this phenotype, no swarming deficiency was recorded for the Δ*rsmA* Δ*toxR* double mutant in PAO1-L in contrast to the PAO1-N Δ*rsmA* strain (Δ*rsmA* and ~59-kb deletion including *toxR*) (Fig. 3). We therefore hypothesised that another genetic element missing in PAO1-N could be responsible for the residual swarming activity in the PAO1-L Δ*rsmA* Δ*toxR* mutant. Hence, alternative genomic modifications explaining this divergence were explored. Among the thirteen distinct SNPs/INDELs found when comparing the two PAO1 sublines, a non-synonymous point mutation was predicted in a PAO1-N gene encoding the c-di-GMP degrading PDE BifA (Supplementary Table 1). This mutation results in tyrosine to aspartate substitution at amino acid 442 (Y442D), a position immediately preceding a glutamine (Q443) involved in substrate binding^53,54^. This suggested that the Y442D substitution in PAO1-N might reduce BifA affinity for its substrate by introducing a negative charge next to the glutamine involved in c-di-GMP binding.

Interestingly, a PAO1 *bifA* mutant was previously shown to have increased intracellular c-di-GMP levels leading to a non-swarming phenotype^22,55,56^. Thus, to determine whether defective BifA activity contributed to the swarming motility defect in PAO1-N Δ*rsmA* and played a role in the differential swarming behaviour between sublines, the motility of strains generated by introducing *bifA*^*Y442D*^ or *bifA*^*D442Y*^ point mutations in PAO1-L WT, PAO1-L Δ*rsmA* Δ*toxR* and PAO1-N Δ*rsmA* were also studied. As shown in Fig. 3, the triple mutation Δ*rsmA* Δ*toxR bifA*^*Y442D*^ resulted in swarming impairment in PAO1-L. At the same time, the reversion of Y442D to D442Y restored swarming motility in PAO1-N Δ*rsmA*, suggesting that *bifA* is the third genetic element involved in this phenotype which potentially explains motility differences between the two PAO1 sublines.

### RsmA positively controls *toxR* expression via PvdS

Given that the alternative sigma factor PvdS is required for optimal *toxR* expression^57^ and that *pvdS* transcript levels were shown to be affected by RsmA^4,58^, we sought to determine whether RsmA regulates ToxR activity partly through the control of *pvdS* expression therefore establishing a link between both regulators and swarming motility. To this end, transcriptional fusions of the *lux* operon to the *toxR* promoter region were constructed using the mini-CTX system^59^ and introduced into the PAO1-L WT and the IPTG-inducible *rsmA*_*ind*_, Δ*pvdS* and Δ*pvdS rsmA*_*ind*_ mutant chromosomes. Moreover, since *toxR* can be transcribed from two promoters (P1 and P2^60^) we investigated the effect of a *rsmA* mutation on expression from the complete intergenic region upstream of *toxR* (*P*_*toxR1,2*_*-luxCDABE*) as well as from each promoter separately (*P*_*toxR1*_/*P*_*toxR2*_ - *luxCDABE*) (Supplementary Fig. 3A). Additionally, since the transcription of *pvdS* is upregulated under iron-limiting conditions^61^, the *toxR* reporter strains were tested under iron deficiency using CAA medium.

As shown in Supplementary Fig. 3B, the non-induced PAO1-L *rsmA*_ind_ strain exhibited lower *toxR* expression levels than WT. Conversely, IPTG-induced expression of *rsmA* in PAO1-L *rsmA*_ind_ stimulated *toxR* expression to levels matching or above those of the WT, suggesting a positive impact of RsmA on ToxR production. Results from assays including Δ*pvdS* and Δ*pvdS rsmA*_*ind*_ strains showed a significant reduction of P2 promoter activity in the Δ*pvdS* mutant compared with WT strain. In agreement with previous studies, P2-driven expression was upregulated under low iron conditions^57^ (Supplementary Fig. 3C). In addition, in the absence of PvdS, RsmA could no longer activate *toxR* expression, suggesting that the RsmA control on *toxR* expression is at the transcriptional level via PvdS (Supplementary Fig. 3C).

### ToxR impacts on the Rhl QS system expression and rhamnolipid biosynthesis

**Fig. 4:**
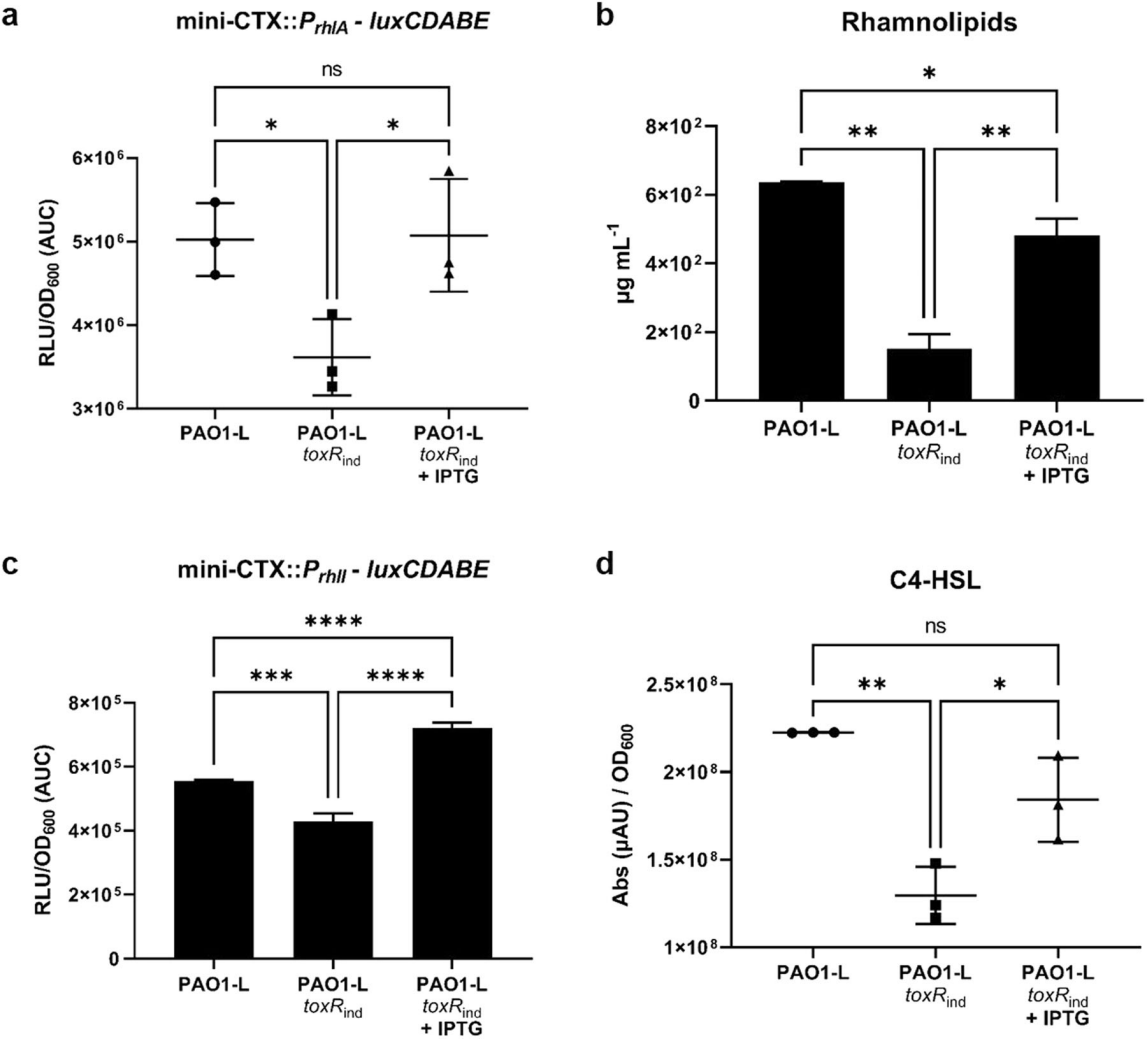
Impact of ToxR on the Rhl system and rhamnolipid production. In all these experiments gene expression and phenotypic analysis were performed in the PAO1-L WT and *toxR*_ind_ mutant strains with and without 1 mM IPTG induction of *toxR*. **a** Transcriptional activity of the *rhlA* promoter assessed at 37 °C in LB by measuring the luminescence emitted by cells containing a *P*_*rhlA*_ - *luxCDABE* transcriptional reporter integrated in the chromosome using the mini-CTX system. Values correspond to the area under the curve (AUC) derived from plotting relative light units normalized to culture density (RLU/OD_600_) over time (24 h). **b** Rhamnolipid production measured using the Orcinol method^86^ from the supernatant fraction of the three strains tested grown in LB at 37 °C for 24 h. **c** Effect of *toxR* mutation on the transcriptional activity of the *rhlI* promoter in LB at 37 °C for 24 h. Values correspond to the area under the curve (AUC) derived from plotting relative light units normalized to culture density (RLU/OD_600_) over time (24 h). **d** C4-HSL UV absorbance values (214 nm) normalised to PAO1-L WT and *toxR*_ind_ mutant growth (OD_600_) in LB at 37 °C for 6 h. C4-HSL was extracted from culture supernatant fractions using acidified ethyl acetate and analysed by LC-MS. Values given are averages from three different cultures ± standard deviation. Statistical differences between group means were determined by one-way ANOVA analysis using Tukey’s multiple comparisons test. (*p < 0.05, **p < 0.01, ***p < 0.005, ****p < 0.0001).

Previous studies have shown that iron depletion stimulates surface motility in *P. aeruginosa*^62,63^. Moreover, rhamnolipid biosynthesis is also upregulated under low iron conditions and is under the control of RhlR^30,35,64^. The correlation between surface motility, iron regulation and the Rhl QS system led us to hypothesise that the mechanism by which ToxR impacts on swarming could be linked to *rhl* expression and hence rhamnolipid production. To investigate this, we first compared expression of the rhamnosyltransferase gene *rhlA* using a P*rhlA* chromosomal transcriptional fusion to the *lux* operon in the PAO1-L WT and the *toxR* conditional IPTG-inducible mutant strain *toxR*_*ind*_ using the integrative vector mini-CTX. A significant reduction in *rhlA* transcription in the *toxR* mutant was observed with respect to the WT strain (Fig. 4a). Conversely, the IPTG-induced expression of *toxR* restored the expression of *rhlA* to WT levels (Fig. 4a), indicating that ToxR has a positive impact on *rhlA* expression. To determine whether the reduced transcription of *rhlA* observed PAO1-L *toxR*_*ind*_ resulted in decreased rhamnolipid production, extraction and quantification of these biosurfactants were performed in both strains. Results showed that rhamnolipid yields were reduced in the *toxR*_*ind*_ strain when compared to WT levels and addition of IPTG stimulated rhamnolipid production (Fig. 4b), confirming the positive impact of ToxR on rhamnolipid biosynthesis.

To establish whether ToxR modulates rhamnolipid production via the activation of the Rhl QS system, the effect of the *toxR* mutation on the expression of *rhlI*, coding for the C4-HSL autoinducer synthase RhlI, was assessed using a mini-CTX*lux* transcriptional fusion of P*rhlI* in PAO1-L *toxR*_*ind*_. In the absence of IPTG (ToxR negative), *toxR*_*ind*_ showed ~25% reduction in *rhlI* expression compared with the WT. Conversely, the addition of IPTG led to an increase of ~25% in *rhlI* expression over the WT levels (Fig. 4c), suggesting a positive effect of ToxR on *rhlI* expression under the conditions tested. We then measured the levels of C4-HSL produced by the WT and *toxR*_*ind*_ strains using LC-MS. The *toxR*_ind_ strain showed reduced levels of C4-HSL and the addition of IPTG restored the levels of this molecule close to those of the WT strain (Fig. 4d), further supporting an impact of ToxR on the Rhl QS system. These results provide further insights into the mechanism by which ToxR affects swarming motility in PAO1 through the activation of rhamnolipid production via the modulation of the Rhl QS system.

### ToxR negatively regulates biofilm formation and Psl/Pel exopolysaccharide production

**Fig. 5:**
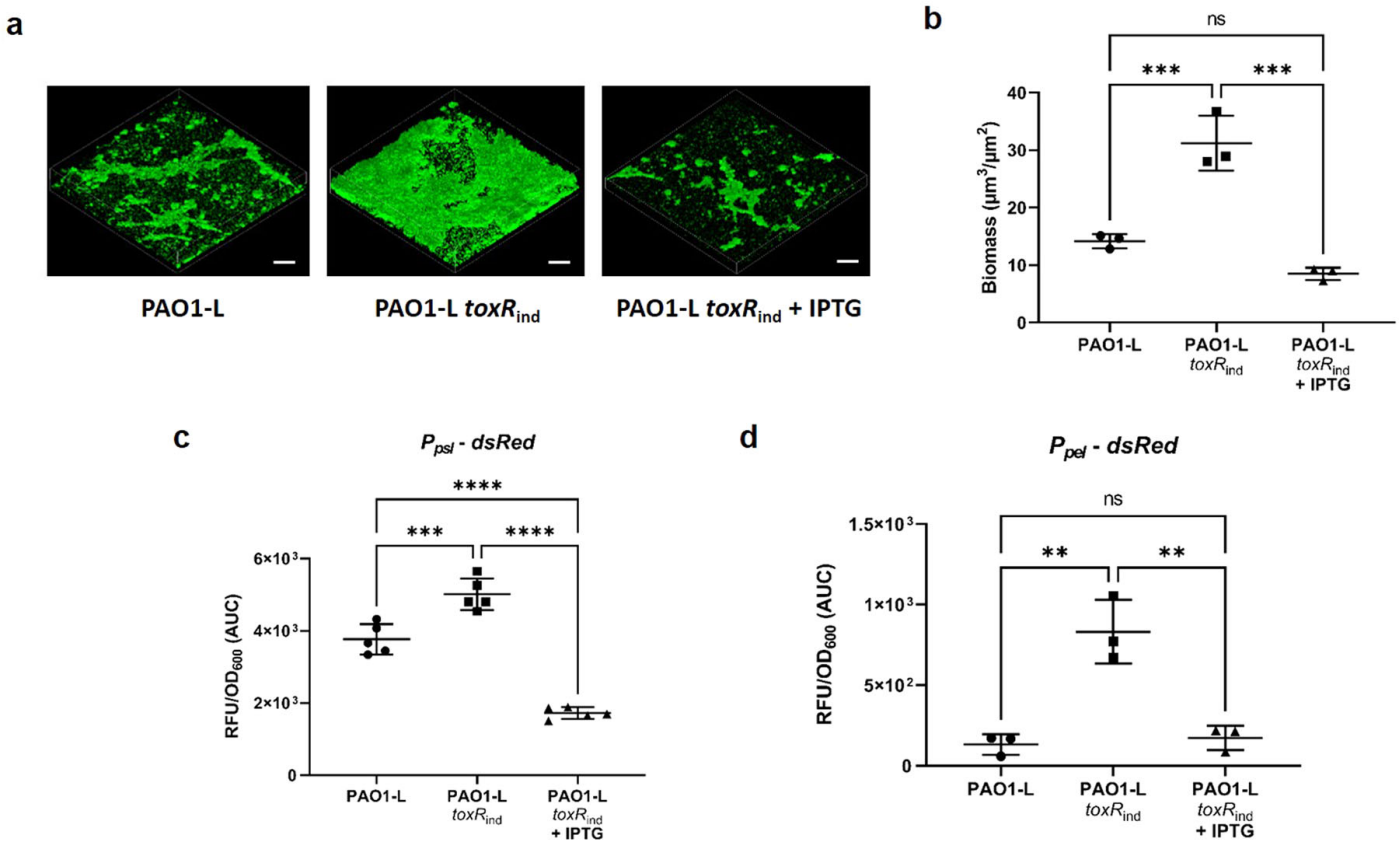
ToxR has a negative impact on biofilm formation and *psl* and *pel* expression. **a** Confocal images of *mClover3*-labelled *P. aeruginosa* PAO1-L WT and *toxR*_ind_ mutant ±1 mM IPTG biofilms obtained after 48 h incubation in microfluidic channels (Bioflux, Fluxion) in RPMI-1640 medium. Scale bar: 100 µM. **b** Quantification of biomass from confocal images of PAO1-L WT and *toxR*_ind_ mutant (±1 mM IPTG) biofilm cultures using Comstat2^89^. **c** and **d** Effect of *toxR* mutation on EPS biosynthetic gene transcription. Expression was determined by measuring the fluorescence from wild type PAO1-L and the *toxR* inducible mutant *toxR*_ind_ cells (±1 mM IPTG) carrying the transcriptional fusions (**c**) *P*_*psl*_*-dsRed* and (**d**) *P*_*pel*_ - *dsRed* in their chromosomes and grown at 37 C for 24 h in LB. Reported values are averages from three different cultures ± SD and correspond to the area under the curve (AUC) derived from plotting relative fluorescent units normalized to culture density (RLU/OD_600_) over time. Statistical differences between group means were determined by one-way ANOVA analysis using Tukey’s multiple comparisons test. (*p < 0.05, **p < 0.01, ***p < 0.005, ****p < 0.0001).

Swarming motility and biofilm formation are intimately interconnected and described as inversely regulated in *P. aeruginosa*^21–23^. Given that ToxR modulates swarming motility in PAO1, we monitored biofilm formation by the *toxR* conditional mutant *toxR*_*ind*_ and PAO1-L WT strains using Bioflux microfluidic chambers to assess whether ToxR could also affect this phenotype. Our results showed that biofilm growth was enhanced in the absence of *toxR* expression, while the IPTG-induced transcription of this gene decreased biofilm biomass compared to the parental PAO1-L WT strain (Fig. 5a, b), suggesting that ToxR has a negative impact on biofilm formation. To study whether changes in exopolysaccharide (EPS) biosynthetic genes expression mediated the effect of ToxR on biofilm formation, the activity of the EPS Psl and Pel biosynthetic operon promoters was determined by measuring the fluorescence from cells containing *P*_*psl*_*-dsRed* and *P*_*pel*_*-dsRed* reporter fusions, respectively. Results showed that the inactivation of *toxR* significantly increased *psl* and *pel* gene expression especially for the *pel* promoter fusion. Conversely, *toxR* complementation through the addition of IPTG reduced the expression of both EPS biosynthetic gene clusters (Fig. 5c, d). These data suggest that ToxR partially represses biofilm formation through a negative impact on EPS production.

### ToxR binds c-di-GMP and negatively affects signalling by this second messenger

**Fig. 6:**
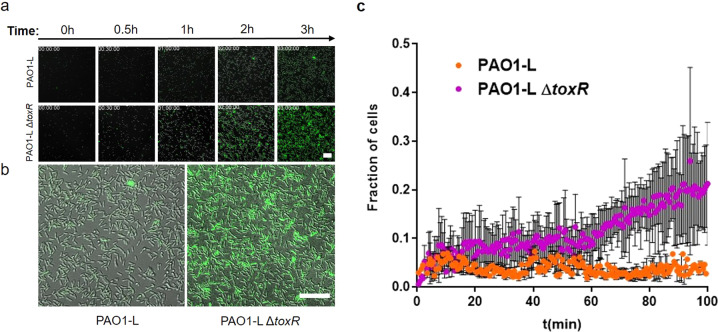
ToxR has a negative impact on *cdrA* expression as an indirect measurement of free c-di-GMP levels. **a** Fluorescence output of P*cdrA gfp*-expressing PAO1-L and Δ*toxR* cells as they transition from liquid culture to glass surface over time (3 h). Scale bar: 20 µm. **b** Representative combined Differential Interference Contrast (DIC) and widefield epifluorescence images of PAO1-L and Δ*toxR* after 3 h exposure to a glass surface. Scale bar: 27 µm. **c** Graph shows the fraction of cells expressing *P*_*cdrA*_*-gfp*(ASV)^C^ over time for the first 100 min after attachment to a glass surface. Dots represent mean ±1 SEM (*n* = 3).

Our results show that ToxR, in addition to regulating the production of exotoxin A, influences swarming and biofilm formation in *P. aeruginosa*. Given that both phenotypes are known to be modulated by c-di-GMP signalling, we hypothesised that ToxR might influence c-di-GMP levels impacting those traits. To test this, the intracellular levels of c-di-GMP were semi-quantified in cells transitioning from motility to sessile lifestyles by monitoring the activity of the transcriptional fusion *P*_*cdrA*_*-gfp*(ASV)^C^, which responds to the levels of this signal^65^, in the PAO1-L WT and Δ*toxR* mutant strain. As shown in Fig. 6 (and Supplementary Fig. 4), the *toxR* mutant displayed high levels of *cdrA* promoter activity, well above that of the WT, consistent with elevated c-di-GMP levels and supporting the hypothesis that ToxR impacts on motility/biofilm formation by reducing c-di-GMP signalling.

**Fig. 7:**
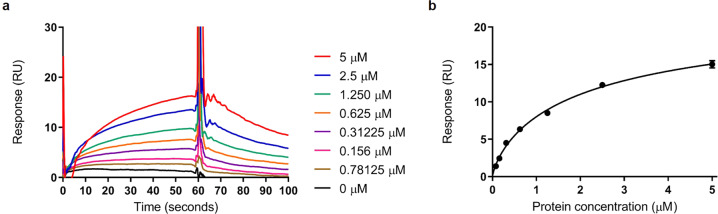
ToxR is a c-di-GMP-binding protein. Purified ToxR was tested for dinucleotide binding by Surface Plasmon Resonance (SPR) as previously described^67^. **a** SPR sensorgrams showing affinity measurements of an increasing range of ToxR concentrations (0–5 µM) binding to biotinylated c-di-GMP. Protein binding and dissociation phases are shown. **b** Affinity fit for ToxR-c-di-GMP binding. The binding response for each concentration was recorded, and the *K*_*D*_ values for ToxR binding to c-di-GMP (2.36 ± 0,7 µM) were calculated using Biacore T200 BiaEvaluation software version 1.0 (GE Healthcare) and confirmed by GraphPad Prism. The experiment was repeated three times independently.

An EAL domain was previously identified in ToxR (Table 1) however, given the absence of conserved residues critical for PDE activity in its sequence (Supplementary Fig. 5), this regulator was assumed to lack c-di-GMP catalytic activity^66^. To verify that the degenerate EAL domain in ToxR is an enzymatically inactive PDE sequence, a 6×His-fusion ToxR protein was expressed, purified and tested for c-di-GMP degradation activity by LC-MS analysis. No c-di-GMP cleavage was detected, indicating that ToxR does not negatively regulate c-di-GMP signalling through degradation of this second messenger. To test whether c-di-GMP is a ToxR ligand, a Surface Plasmon Resonance (SPR) binding assay using purified ToxR protein was performed as previously described^67^. As shown in Fig. 7, we were able to detect reproducible concentration-dependent binding of c-di-GMP by ToxR, with a calculated K_D_ of 2.36 ± 0.7 μΜ. This indicates that ToxR is a c-di-GMP-binding protein and suggests that ToxR may impact on swarming and biofilm formation through direct stimulation of additional enzymes able to metabolise this signalling molecule explaining the altered c-di-GMP levels in the *toxR* mutant.

## Discussion

An analysis of the genetic differences between *P. aeruginosa* PAO1-Lausanne and PAO1-Nottingham sublines, combined with an exhaustive screening for genes able to complement swarming in PAO1-N Δ*rsmA* mutant using a plasmid library containing random chromosomal fragments of PAO1-L, allowed us to identify new genetic elements restoring this motility. The robustness of our approach is supported by the finding of genes previously described to be involved in swarming i.e. *bifA* and *rsmN*^22,46^, but also ORFs encoding proteins with conserved domains implicated in c-di-GMP metabolism and hence expected to have an impact on this surface-associated motility^15,22,23,68^. While seven potential genes able to restore a swarming deficiency in PAO1-N Δ*rsmA* were identified, we have not yet characterised these further and focused on *toxR*, currently known to encode a regulator of ETA biosynthesis in *P. aeruginosa*^44^.

Remarkably, the *P. aeruginosa* PAO1-N subline used in our laboratory, and which exhibits a reduction in swarming motility compared to PAO1-Lausanne, contains a substantial deletion in its chromosome including the regulatory gene *toxR*. This 59-kb deletion was found to be present in strain PANO67, the first *P. aeruginosa* mutant selected in our laboratory^69^, suggesting that it was already present in the PAO1 subline that we obtained over 30 years ago, but the exact origin of which can no longer be ascertained. Additionally, a non-synonymous point mutation in *bifA*, encoding a c-di-GMP PDE with known roles in motility and early biofilm formation^22^, was identified in PAO1-N that could potentially affect substrate binding by this enzyme. Although mutation of *toxR* or *bifA* causes only a partial swarming defect in PAO1-L, here we show that a ToxR deficiency in combination with that of the regulatory proteins RsmA and BifA leads to total swarming impairment in this PAO1 subline. Furthermore, RsmN was found to bind the *bifA* transcript before^3^ suggesting a link between these two factors influencing swarming.

Our results indicate that ToxR affects swarming by stimulating expression of the autoinducer synthase RhlI and subsequently increasing the levels of the signal C4-HSL and the rhamnolipid surfactants, which support migration by lowering surface tension thus facilitating flagella-based propulsion^28^. Interestingly, a previous study reported that low levels of c-di-GMP induce the expression of the Rhl QS operon, and this finding correlated with an increased production of Rhl-regulated virulence factors including rhamnolipids^70^. Moreover, our results show that ToxR has an impact on biofilm formation, a phenotype closely associated with chronic infections. This control could be exerted, at least partially, through repression of Psl and Pel production as we observed that ToxR negatively impacts on the expression of the biosynthetic genes for these exopolysaccharides. Regulation of Psl and Pel has been shown to be controlled by c-di-GMP through the activity of PDEs and DGCs^47,71^. Given these factors and the fact that c-di-GMP modulates the transition from motile to sessile (biofilm) lifestyles in *P. aeruginosa*^71^, it seems reasonable that ToxR could indirectly exert its effect on these processes through governing c-di-GMP levels in the cell.

Despite the prediction that ToxR incorporates a single putative EAL domain, an ubiquitous motif linked to PDEs and c-di-GMP turnover in bacteria^72^, we could not detect c-di-GMP-specific PDE activity when testing the purified protein in vitro. Consistent with this result, ToxR was previously defined as a stand-alone EAL domain protein and expected to lack catalytic activity against c-di-GMP due to the absence of extra domains assisting dimerisation and lack of conserved residues essential for c-di-GMP metabolism^54,66^. Although catalytically inactive, EAL domain proteins can retain the ability to bind c-di-GMP and therefore additional interaction partners with these proteins are anticipated^66,73^. Indeed, our results show that ToxR binds c-di-GMP with high affinity and the intracellular levels of this second messenger increase significantly in a *toxR* mutant compared with the WT strain, suggesting that the ToxR regulon may contain additional c-di-GMP-metabolizing enzymes responsible for ultimately controlling the levels of this intracellular signal leading to the changes in gene expression and the phenotypes observed. Therefore, identification of the residues within ToxR that are involved in c-di-GMP binding could form part of future studies. In particular, it will be interesting to see if a *toxR* mutant in the EXLXR motif is still able to bind c-di-GMP and influence surface-associated behaviour.

**Fig. 8:**
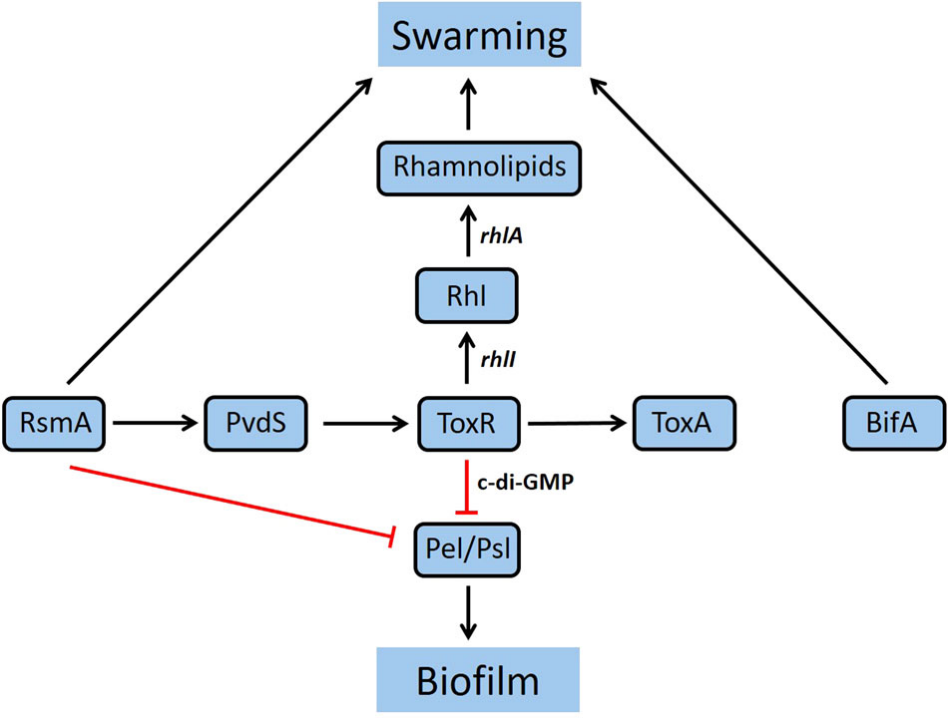
Proposed regulatory pathway involving ToxR, RsmA and PvdS controlling *P. aeruginosa* swarming and biofilm formation. Elements acting positively or negatively are represented by arrowheads and bars, respectively.

Notably, iron depletion, an environmental parameter that stimulates *toxR* expression^57^, represses biofilm formation and promotes biosynthesis of the pyoverdine siderophore necessary for acute infections^41^ are conditions where low levels of c-di-GMP are expected. In addition, we show that RsmA exerts positive control over ToxR via PvdS, strengthening the established links between the Rsm and c-di-GMP regulatory networks^74^ (Fig. 8). The positive impact of RsmA on *pvdS* expression under iron limiting conditions found here supports the results of Brencic and Lory^4^ who showed a reduction in *pvdS* transcript levels in an *rsmA* mutant compared with the WT *P. aeruginosa* PAK strain and suggests that this regulation is conserved across different strains. The link between RsmA, PvdS, ToxR and c-di-GMP signalling is further supported by previous work from Jones et al.^75^ in which the authors demonstrated that mutants in AmrZ, a transcriptional factor important for *P. aeruginosa* virulence accumulates intracellular c-di-GMP, and AmrZ increased expression of RsmA (1.55 fold) and repressed many genes involved in iron acquisition including PvdS (1.65 fold) and ToxR (1.5 fold)^75^.

Previous attempts to determine the mechanism by which ToxR activates ETA production have found that ToxR can function with the RNA polymerase of *E. coli* and *P. aeruginosa* to efficiently transcribe *toxA* both in vitro and in vivo and the ToxR-mediated enhancement of *toxA* expression is improved in the presence of cytoplasmic extracts of *Pseudomonas aeruginosa*^76,77^. Moreover, ToxR has been shown to be required for open-complex formation at the *toxA* promoter^77^. Interestingly, several studies have documented that c-di-GMP can control the switch between acute and chronic infection modes in bacteria by facilitating protein-protein and protein-DNA interactions^72^. Therefore, it is tempting to hypothesise that c-di-GMP could facilitate ToxR regulation of transcription, for instance through modulation of ToxR stability, as it has been reported that c-di-GMP binding may lead to enhanced stability of PgaCD interaction in *E. coli*^78^. However, given that EAL proteins have not been shown to bind DNA to date but are known to bind other proteins, stimulation of another regulatory protein or a ToxR-RNA polymerase interaction seems to be a more likely model for ToxR function than direct transcriptional control.

Overall, the work presented here shows that ToxR, originally thought to exclusively activate ETA production, has a negative impact on biofilm formation through the modulation of Psl and Pel exopolysaccharides, and a positive impact on swarming through activation of the Rhl QS system and hence rhamnolipid production. This regulation is also modulated by the post-transcriptional regulator RsmA via PvdS and hence iron depletion. However, the molecular basis by which ToxR impacts on these regulatory networks through c-di-GMP binding remains to be elucidated.

## Methods

### Bacterial strains and culture conditions

Strains, plasmids and oligonucleotides used in this study are listed in Supplementary Table 3. *E. coli* and *P. aeruginosa* strains were routinely grown at 37 °C on lysogeny broth (LB) or LB agar supplemented with antibiotics as required. For studying *toxR* expression, *toxR* reporter strains were grown under iron deficiency using CAA medium^79^. IPTG was added to cultures at 1 mM final concentration to complement inducible mutant strains. Swarming assays were performed in 60 ×15 mm dishes with 8 g/L Nutrient Broth No.2 (Oxoid), 0.5% w/v Bacto agar (Difco) and 0.5% w/v D-glucose (Sigma). Plates were inoculated with 3 µL of OD_600_ 1.0 ON cultures washed in fresh LB. Plates were then incubated for 16 h at 37 °C.

Biofilms were grown continuously in microfluidic channels using a Bioflux system (Fluxion). Briefly, mClover3-labelled^80^ *P. aeruginosa* PAO1-L WT and *toxR*_ind_ mutant strains were grown at 37 °C for 16 h in 2 mL Lysogeny broth (LB) broth. The cultures were diluted to OD_600_ 0.05 in RPMI-1460 (Lonza) and used to seed the microfluidic channels. The biofilm was allowed to form at a flow rate of 16 μL/h (corresponding to a shear rate of 0.5 dyn/cm^2^) at a temperature of 37 °C for a period of 48 h in RPMI-1640 (Lonza Bioscience).

### *P. aeruginosa* subline PAO1-L genomic library

To find genetic elements in PAO1-L restoring swarming motility in *P. aeruginosa* PAO1-N Δ*rsmA*, a genomic library was generated by partially digesting the PAO1-L chromosome with Sau3AI and 2–4 kb fragments cloned into pME6000 plasmid. The resulting plasmids were transformed into *P. aeruginosa* PAO1-N Δ*rsmA* and each clone screened for restoration of swarming^46^.

### Sequencing of *P. aeruginosa* PAO1 sublines and optical restriction genome mapping

To catalogue single nucleotide polymorphisms (SNPs) and small insertions/deletions (INDELs) in PAO1-L and PAO1-N sublines, paired-end sequencing libraries were prepared using the Illumina Nextera kit according to the manufacturer’s protocols (Illumina), and whole-genome sequencing was performed on an Illumina MiSeq platform. Whole-genome sequence datasets were subjected to adaptor trimming using Trimmomatic version 0.30^80^ and hard trimming at the 5′ end using a custom Perl script. Trimmed reads were aligned to the modified PAO1-UW sequence^3^ using Smalt version 0.7.0.1 (sanger.ac.uk/tool/smalt-0/) with a kmer of 12 and a step size of 2. The alignment files were sorted, and PCR duplicates were removed using Samtools^81^. INDEL realignment was carried out with the Genome Analysis Toolkit^82^, and SNPs and INDELs were determined using Samtools mpileup, employing varying base quality thresholds (parameter-Q). SNPs were filtered to exclude those with low coverage (*<*5x) and those not supported by at least two reads on each strand using a custom Perl script. A custom Perl script was used to characterise SNPs in genes, and the resulting amino acid changes. Regions with zero read coverage were detected using the “genomeCoverageBed” functionality of bedtools with the -bga option (version 2.17.0)^83^. *De novo* assembly was carried out using Velvet version 1.2. with a kmer of 121 with no scaffolding and the expected coverage flag set to auto.

Large scale chromosomal rearrangements, deletions and insertions in sublines PAO1-L and PAO1-N were analysed by optical restriction mapping^50,51^. Briefly, high molecular weight (HMW) DNA was isolated from single colony cultures of *P. aeruginosa* PAO1-L and PAO1-N using the HMW DNA Isolation Kit (Opgen Inc.) according to the manufacturer’s instructions. The quality of HMW DNA was assessed using an Argus QCard Kit (Opgen Inc.), and the concentration of the HMW DNA was adjusted using dilution buffer. DNA molecules were stretched and linearised on charged glass coverslips using a high-density channel-forming device (CFD). The restriction endonuclease BamHI was used in an Argus MapCard (Opgen Inc), and the glass coverslips were incubated in a MapCard Processor to allow digestion of the DNA molecules on the glass surface. The fragmented DNA was then stained with intercalating fluorescent dye, and the DNA molecules were imaged under a high-resolution microscope. DNA molecules were assembled into a whole-genome map using the OpGen Genome-Builder software. Optical maps were aligned to the reference *P. aeruginosa* subline PAO1-UW^49^ (GenBank accession NC_002516) using the OpGen MapSolver software and visually inspected to locate regions of difference >2 kb, the lower limit for efficient optical mapping.

### Mutant strain construction

In-frame deletion mutants were obtained by two-step allelic exchange using pDM4 or pME3087 vectors. To construct *P. aeruginosa* PAO1-L Δ*rsmA*, Δ*toxR* and Δ*pvdS* mutants, two PCR products amplifying each gene’s upstream and downstream nucleotide regions were generated using the primer pairs D1FW/D1RV and D2FW/D2RV, respectively (Supplementary Table 3). PCR products were ligated together in pBluescript II KS^+^ to create a deletion in the corresponding gene and subsequently cloned into the suicide vector pDM4 or pME3087 resulting in plasmids pZH13, pJD22 and pJD93, respectively. Following transformation into the target strain by conjugation, single crossovers were selected on chloramphenicol (Cm, 375 μg mL^−1^) or tetracycline (Tc, 125 μg mL^−1^). To achieve *rsmA* and *toxR* deletion, recombinants resistant to Cm were grown in LB supplemented with 10% sucrose overnight and plated on LB-10% sucrose. The carbenicillin enrichment method selected the *pvdS* double-crossover mutants^84^. The resulting *P. aeruginosa* colonies were screened for the loss of antibiotic resistance by plating on LB supplemented with or without Cm/Tc. PCR and sequence analysis confirmed the in-frame deletions.

The conditional mutant IPTG-inducible *toxR* (*toxR*_*ind*_) strain was constructed by introducing the *lacI*^*Q*^ repressor gene and the *tac* promoter transcribing the *lacZ* 5′ untranslated region and its ribosome binding site (RBS) directly upstream of the *toxR* open reading frame, resulting in constitutive transcription and translation only in the presence of IPTG. The suicide plasmid pAMP5 was constructed in several stages and is a pDM4 derivative that carries, in sequential order, the following elements: (a) a 365 bp HindIII-EcoRI fragment of the upstream region of *toxR* obtained by PCR using primers *toxRp*FW/RV (Supplementary Table 3); (b) the 2.0 kb BamHI Sm/Spc integron from pHP45Ω; (c) the 1.5 kb BamHI-EcoRI *lacI*^*Q*^ P*tac* inducible promoter fragment of pME6032 and (d) an 0.5 kb EcoRI-XhoI fragment carrying the *toxR* open reading frame (obtained from pBluescript II KS^+^-based vector constructed for the Δ*toxR* mutant). The mutant was obtained by double crossover of the fragment carried by pAMP5 in *P. aeruginosa* PAO1-L and selection of sucrose and streptomycin-resistant clones.

To generate the *bifA*^*Y442D*^ mutant in PAO1-L and derivative mutants as well as to revert the *bifA* point mutation in PAO1-N, the *bifA* gene was amplified from PAO1-N and PAO1-L genomes using the primer pair BifAFW/BifARS (Supplementary Table 3). The resulting PCR products were ligated with pBlueScript II KS^+^ and subsequently cloned as HindIII-EcoRI fragments into the suicide plasmid pME3087, generating the plasmids pJL103 and pJL108 and used for double crossover recombination. The resulting *P. aeruginosa* colonies were screened for the loss of antibiotic resistance by plating on LB supplemented with or without Tc. PCR and sequence analysis confirmed the mutations.

### Transcriptional reporter fusions

The promoter regions of *toxR*, *rhlA* and *rhlI* were amplified from the PAO1-L genome using the primer pairs ToxRp1FW/RV, ToxRp2FW/RV, ToxRp12FW/RV, RhlApFW/RV and RhlIpFW/RV (Supplementary Table 3). The resulting PCR products were ligated into the mini-CTX*lux* vector using the appropriate restriction enzyme to generate the transcriptional reporters pAMP11 (mini-CTX::*P*_*toxR1*_ - *luxCDABE*), pAMP12 (mini-CTX::*P*_*toxR2*_ - *luxCDABE*), pAMP13 (mini-CTX::*P*_*toxR1,2*_ - *luxCDABE*), pSH30 (mini-CTX::*P*_*rhlA*_ - *luxCDABE*) and pSH32 (mini-CTX::*P*_*rhlI*_ - *luxCDABE*). Finally, the transcriptional reporters were integrated into the chromosome of *P. aeruginosa* PAO1 strains by conjugation with *E. coli* S17.1 λpir followed by selection with Tc.

For *P*_*pel*_ - *dsRed* and *P*_*psl*_*-dsRed* transcriptional reporter construction, the promoter regions of *pelA* and *pslA* genes were amplified by PCR from *P. aeruginosa* PAO1-L chromosomal DNA using primer pairs P*pelAdsRed*FW/RV and P*pslAdsRed*FW/RV, respectively. The reporter *dsRed-express2* gene was amplified using primer pairs *dsRed*FW/RW from pCMVDsRed-Express2 vector and cloned into pBluescript II KS^+^, resulting in plasmid pSC581. Then *dsRed-express2* cut from pSC581 with HindIII/EcoRI, and the KpnI/HindIII cut *pelA* or *pslA* promoter regions were ligated into pUCP18, resulting in plasmids pSC855 and pSC856.

Clones with active fusions were selected and analysed for bioluminescence or fluorescence output activity over growth in CAA or LB media at 37 °C using a 96-well plate TECAN Genios Pro multifunction microplate reader.

### C4-HSL and rhamnolipid quantification

*P. aeruginosa* strains were grown for 6 h (early stationary phase) in LB broth at 37 °C with vigorous shaking (200 rpm). QS molecules were extracted from 5 mL culture by adding 5 mL of acidified ethyl acetate^85^. Sample separation was performed on an Agilent 1200 series HPLC with an Ascentis Express C18 150 ×2.1 mm internal diameter, 2.7 mm particle size, maintained at 50 °C. The mobile phase consisted of formic acid 0.1% (v/v) in water and formic acid 0.1% (v/v) in acetonitrile run as a gradient over 20 min at a flow rate of 0.3 ml min^−1^. C4-HSL identification was performed using a Bruker HCT Plus ion trap LC-mass spectrometer and Hystar software (Bruker). Retention times and MS/MS peak spectra were matched to 10 mM synthetic C4-HSL standard. For rhamnolipids quantification strains were grown at 37 °C in 50-ml Erlenmeyer flasks containing 10 ml of M9 medium supplemented with glycerol (2% vol/vol), glutamate (0.05%), and Triton X-100 (0.05%) with shaking for 18 h. Rhamnolipids were extracted with three volumes of diethyl ether from culture supernatants filtered through a 0.22-μm-pore-size membrane. Extracts were extracted once with 20 mM HCl, and the ether phase was evaporated to dryness. The residue was dissolved in water. Rhamnose content in each sample was determined by duplicate oricinol assays compared to rhamnose standards. Rhamnolipid was determined by the relation that 1.0 mg of rhamnose corresponds to 2.5 mg of rhamnolipid^11,86^.

### ToxR purification

The pBAD TOPO TA expression system (Invitrogen) was used to produce His-tagged ToxR protein within host *E. coli* BL21 (DE3) cells. The *toxR* gene was amplified from PAO1-L genome using primers ToxRtopoFW/RV and cloned into pBAD TOPO vector according to manufacturer’s instructions. *E. coli* BL21 (DE3) cells carrying the resulting pAMP14 vector were cultured overnight in LB at 37 °C with vigorous shaking (200 rpm), diluted 1:100 in Terrific Broth (TB) and grown to OD_600_ 0.4 at 37 °C before protein expression was induced overnight with 0.2% L-Arabinose at 18 °C. Cells were then lysed by sonication and centrifuged at 15,000 × *g* for 1 h. ToxR was purified from the supernatant by NTA-Ni chromatography. HiTrap chelating HP columns of 1 mL (GE healthcare, life sciences) were equilibrated with 10 volumes of washing buffer (20 mM HEPES pH 7.5, 250 mM NaCl, 2 mM MgCl_2_, and 2.5% (v/v) glycerol pH 6.8) and loaded with cell lysate. Following protein immobilisation, the column was washed with 10 volumes of buffer containing 50 mM imidazole before the protein was eluted using 500 mM imidazole buffer in a single step elution. Due to ToxR instability, it was required that later assays were performed within a few hours after protein purification.

### ToxR phosphodiesterase activity and c-di-GMP binding detection methods

To assess cleavage of c-di-GMP by a possible PDE activity of ToxR, purified ToxR (0.7 mg) was incubated with c-di-GMP (100 μM) (Biolog Life Science Institute) for 2 h at 30 °C. The remaining c-di-GMP in the samples was detected by liquid chromatography-mass spectrometry (LC-MS) analysis using the TSQ Quantum Access Max system (Thermo Scientific) in positive-ion mode, as previously described^87^. The areas of the major MS-MS fragments of c-di-GMP (m/z 152 and 540) were measured, and a standard curve was established using defined concentrations of c-di-GMP to allow sample quantification. Moreover, the main degradation product of c-di-GMP cleavage, 5′-phosphoguanylyl-(3′→5′)-guanosine (pGpG), was also quantified in the samples by measuring the area of the major fragment (*m/z* = 152) of the isolated precursor ion (*m/z* = 709) corresponding to pGpG as determined using a standard (Biolog).

To test whether c-di-GMP is in fact a ToxR ligand, a Surface Plasmon Resonance (SPR) binding assay using purified ToxR protein was performed^67^. Briefly, a Biacore T200 system (GE Healthcare) equipped with a Streptavidin SA sensor chip (GE healthcare), containing four flow cells with a SA pre-immobilized to a carboxymethylated dextran matrix each, was used. Flow cell one (FC1) and flow cell three (FC3) were kept blank for reference subtraction. The chip was washed three times with 1 M NaCl and 50 mM NaOH to remove any unconjugated streptavidin. Biotinylated c-di-GMP (100 nM) (BioLog) was immobilised on FC2 and FC4 of the streptavidin chip at a 50 RU immobilisation level with a flow rate of 5 μL/min. Soluble ToxR protein was prepared in SPR buffer (10 mM HEPES, 500 mM NaCl, 0.1% (v/v) Tween 20, 2 mM MgCl_2_, pH 6.8). Samples were injected with a flow rate of 5 μL/min over the reference and c-di-GMP cells for 60 s, followed by buffer flow for 60 s. The chip was washed at the end of each cycle with 1 M NaCl. An increasing range of protein concentrations (78.125 nM, 156.25 nM, 312.5 nM, 625 nM, 1.25 µM, 2.5 µM, 5.0 µM) was used, with replicates for each protein concentration included as appropriate. All sensorgrams were analysed using Biacore T200 BiaEvaluation software version 1.0 (GE Healthcare). The experiment was repeated three times independently.

### Microscopy and image analysis methods

*P. aeruginosa* PAO1-L WT and Δ*toxR* mutant strains carrying the plasmid *P*_*cdrA*_*-gfp*(ASV)^C^ ^65^ were used to report c-di-GMP levels in cells transitioning from liquid culture to a glass surface over time (3 h). For time-lapse imaging, a bespoke multimode microscope (Cairn Ltd)^88^, based in an inverted Eclipse Ti microscope (Nikon), was used. The microscope was fitted with an environmental chamber (Okolab) to regulate temperature and relative humidity. Differential Interference Contrast (DIC) and widefield epifluorescence imaging were carried out using a single channel white MonoLED light source (Cairn Ltd) and a 40× objective (Nikon, CFI Plan Fluor 40×/1.3). Images were acquired using an Orca-Flash 4.0 digital CMOS camera (Hamamatsu) every 30 sec per hour. Experiments were conducted using a 35 mm glass-bottom dish (Cellvis). The fraction of cells expressing *P*_*cdrA*_*-gfp*(ASV)^C^ was calculated by counting objects in each frame in both DIC and epifluorescence mode. Images were processed in ImageJ (NIH), and an automatic threshold was applied using the Yen method to each data set. Bacterial tracks were processed through a custom single-cell tracking algorithm^88^ to determine the average fluorescence (F values) of tracks corresponding to *P*_*cdrA*_*-gfp*(ASV)^C^ expression levels.

Biofilms expressing the fluorescent protein mClover3 were visualised under a Confocal Laser Scanning Microscope (LSM700, Carl Zeiss) using 488 nm laser. At least 5 replicate Z-stack biofilm images were taken randomly, and biomass was quantified using Comstat2 plugin^89^ in ImageJ software.

### Statistical analysis

One-way ANOVA analysis using Tukey’s posthoc multiple comparisons tests were applied to determine whether mutants’ responses differed significantly from that of the parental strain (*p* < 0.05) when compared with the variations within the replicates (*n* = 3) using GraphPad Prism 8.0 (GraphPad Software, Inc., San Diego, CA).

## Supplementary information

Supplementary Information

## Data Availability

Whole genome sequencing results reported in this paper have been deposited at the National Center for Biotechnology Information (NCBI), under BioProject No. PRJNA830884. Assembled genome sequences for sublines PAO1-L and PAO1-N are available from GenBank, Accession Nos. CP099798 and CP099797, respectively. The data that support the findings of this study are available from the corresponding author, MC, upon reasonable request.
