# Supplementary material for: ToxR is a c-di-GMP binding protein that modulates surface-associated behaviour in Pseudomonas aeruginosa
Source: NPJ Biofilms Microbiomes. 2022 Aug 18;8:64. doi: 10.1038/s41522-022-00325-9 (PMC9388670; doi:10.1038/s41522-022-00325-9)
Supplement: Supplementary file 1 — Supplementary Information [file 41522_2022_325_MOESM1_ESM.pdf]

Supplementary Section

ToxR is a c-di-GMP binding protein that modulates surface-associated behaviour in *Pseudomonas aeruginosa*.

Jean-Frédéric Dubern[1*], Manuel Romero[1*], Anne Mai-Prochnow[1, 2], Marco Messina[1, 3], Eleftheria Trampari[4, 5], Hardeep Naghra-van Gijzel[1, 6], Kok-Gan Chan[7,8], Alessandro M. Carabelli[9, 10], Nicolas Barraud[11, 12], James Lazenby[1, 5], Ye Chen[1,13], Shaun Robertson[1], Jacob Malone[4,14], Paul Williams[1], Stephan Heeb[1] and Miguel Cámara[1‡]

[1]National Biofilms Innovation Centre, Biodiscovery Institute and School of Life Sciences, University of Nottingham, Nottingham, UK.

[4]Department of Molecular Microbiology, John Innes Centre, Norwich, UK.

[7]Institute of Biological Sciences, Faculty of Science, University of Malaya, Kuala Lumpur, Malaysia.

[8]International Genome Centre, Jiangsu University, Zhenjiang, China.

[9]School of Pharmacy, Boots Science Building, University of Nottingham, Nottingham, UK.

[11]Centre for Marine Bio-Innovation, School of Biotechnology and Biomolecular Science, University of New South Wales, Sydney, Australia.

[14]School of Biological Sciences, University of East Anglia, Norwich, UK.

[2] Present address: School of Chemical and Biomolecular Engineering, University of Sydney, Australia.

[3] Present address: Department of Science, University Roma Tre, Rome, Italy.
[5]Present address: Quadram Institute Bioscience, Norwich Research Park, Norwich, Norfolk, UK.
[6]Present address: Genomic Sciences, GlaxoSmithKline Research and Development, Stevenage, UK.
[10]Present address: Department of Medicine, University of Cambridge, Cambridge, UK.
[12]Present address: Genetics of Biofilms Unit, Institut Pasteur, Paris, France.
[13]Present address: Q Squared Solutions, Crystal Plaza, Pudong, Shanghai, China.

*These authors contributed equally to this work
‡Corresponding author: miguel.camara@nottingham.ac.uk

SUPPLEMENTARY TABLES

Supplementary Table 1. Single nucleotide polymorphisms (SNPs) and small insertions/deletions (INDELs) present in the genomic sequences of PAO1-L and PAO1-N sublines when compared to the reference genome of PAO1-UW[1].

Locus ID	Gene name	SNPs/INDELs	Description	Subline
PA0373	*ftsY*	GCCGAGC	6-bp insertion, in-frame insertion of glutamic acid-poline (EP) to a stretch of 8 EP repeats	PAO1-N
PA0683	*hxcY*	GC	1-bp insertion, frameshift at codon 73 (out of 382)	PAO1-L
PA1843	*metH*	A	GTc to GTt, silent V547V	PAO1-N
PA2492	*mexT*	-	1-bp deletion, frameshift at codon 83 (out of 348)	PAO1-N
PA2494	*mexF*	G	TAc to Tag, Y326* premature translation termination (1,062 codons in total)	PAO1-N
PA3578	-	A	Agc to Agt, silent S65S	PAO1-N
-	-	G	Intergenic PA3969-*amn* region, downstream of both	PAO1-L
-	-	C	Intergenic PA3969-*amn* region, downstream of both	PAO1-L
PA4367	*bifA*	G	tAC to gAC, Y442D substitution (687 codons in total)	PAO1-N
PA4498	*mdpA*	CGGTGC	5-bp insertion, frameshift at codon 333 (out of 406)	PAO1-N
PA4514	*piuA*	-	1-bp insertion, frameshift at codon 311 (out of 754)	PAO1-N
PA4525	*pilA*	C	aGC to gGC, S64G substitution (150 codons in total)	PAO1-N
PA5100	*hutU*	GC	Acg to Agc, T431S substitution (560 codons in total)	PAO1-L

Supplementary Table 2. Genetic elements missing in PAO1-N as part of the ~59-kb deletion

Locus ID	Name	Description
PA0707	*toxR*	ToxR/RegA protein regulating exotoxin A production. Deletion starts 1 bp before start codon.
PA0706	*cat*	chloramphenicol acetyltransferase, antibiotic resistance gene
PA0705	*migA*	alpha-1,6-rhamnosyltransferase MigA, possibly involved in lipopolysaccharide biosynthesis
PA0704	*gatA*	putative aspartyl/glutamyl-tRNA amidotransferase subunit A
PA0703	-	probable major facilitator superfamily (MFS) transporter
PA0702	-	hypothetical protein similar to sterol desaturase/fatty acid hydroxylase
PA0701.1	-	probable AraC-like transcriptional regulator (unannotated)
PA0701	-	probable LysR-like transcriptional regulator
PA0700	-	hypothetical protein of unknown function
PA0699	-	probable peptidyl-prolyl isomerase (chaperones & heat shock protein family)
PA0698	-	hypothetical protein of unknown function
PA0697	-	hypothetical protein of unknown function
PA0696	-	hypothetical protein of unknown function
PA0695	-	probable TonB-like membrane-linking protein
PA0694	*exbD2*	predicted biopolymer transport protein ExbD2
PA0693	*exbB2*	predicted biopolymer transport protein ExbB2
PA0692	*pdtB*	phosphate depletion regulated TPS partner B
PA0691	*phdA*	prevent host death protein A
PA0690	*pdtA*	phosphate depletion regulated TPS partner A
PA0689	*lapB*	low-molecular-weight alkaline phosphatase B
PA0688	*lapA*	low-molecular-weight alkaline phosphatase A
PA0687	*hxcS*	type II secretion system protein involved in alkaline phosphatase secretion
PA0686	*hxcR*	type II secretion system protein involved in alkaline phosphatase secretion
PA0685	*hxcQ*	type II secretion system protein involved in alkaline phosphatase secretion
PA0684	*hxcZ*	type II secretion system protein involved in alkaline phosphatase secretion

PA0683	*hxcY*	type II secretion system protein involved in alkaline phosphatase secretion
PA0682	*hxcX*	type II secretion system protein involved in alkaline phosphatase secretion
PA0681	*hxcT*	type II secretion system protein involved in alkaline phosphatase secretion
PA0680	*hxcV*	type II secretion system protein involved in alkaline phosphatase secretion
PA0679	*hxcP*	type II secretion system protein involved in alkaline phosphatase secretion
PA0678	*hxcU*	type II secretion system protein involved in alkaline phosphatase secretion
PA0677	*hxcW*	type II secretion system protein involved in alkaline phosphatase secretion
PA0676	*vreR*	sigma factor regulator VreR, PUMA3 cell-surface signaling system
PA0675	*vreI*	ECF sigma factor VreI, PUMA3 cell-surface signaling system
PA0674	*vreA*	TonB-like ECF receptor VreA, PUMA3 cell-surface signaling system
PA0673	-	hypothetical protein of unknown function
PA0672	*hemO*	predicted heme oxygenase HemO
PA0671	-	probable SOS-response cell division inhibitor
PA0670	-	probable DNA Polymerase Y-family protein
PA0669	-	Probable DNA polymerase alpha chain
PA4280.1	-	5S ribosomal RNA subunit, identical to PA0668.5
PA4280.2	-	23S ribosomal RNA subunit, identical to PA0668.4 but 2 bp. smaller
PA4280.3	-	tRNA-Ala, identical to PA PA0668.3
PA4280.4	-	tRNA-Ile, identical to PA0668.2
PA4280.5	-	16S ribosomal RNA, 1536 bp long, identical to PA0668.1 Deletion starts 308 bp after transcriptional start site

Supplementary Table 3. Bacterial strains, plasmids and oligonucleotides used in this study.

Strain, plasmid or oligonucleotide	Relevant characteristics	Reference or origin
**Strain**		
<u>*P. aeruginosa*</u>		
PAO1-N	Nottingham collection wild type *P. aeruginosa* strain	Holloway collection, source unknown
PAO1-L	Lausanne collection wild type *P. aeruginosa* strain	Holloway collection, via D. Haas
PAZH13-N	*rsmA* in frame deletion mutant, derivative of PAO1-N	[2]
PAZH13-L	*rsmA* in frame deletion mutant, derivative of PAO1-L	This study
PASK10-L	*rsmA*::Sm/Spc-*lacIQ*-Ptac-*rsmA*; IPTG-inducible, conditional *rsmA* mutant of PAO1-L	[3]
PAAMP2	*toxR*::Sm/Spc-*lacIQ*-Ptac-*toxR*; IPTG-inducible, conditional *toxR* mutant, obtained by allelic exchange using pAMP5 on PAO1-L	This study
PAJD55	*toxR* in frame deletion mutant, obtained by allelic exchange using pJD22 on PAO1-L	This study
PAJD56	*pvdS* in frame deletion mutant, obtained by allelic exchange using pJD93 on PAO1-L	This study
PAJD58	*toxR* in frame deletion mutant, obtained by allelic exchange using pJD22 on PAZH13-L	This study
PAJD173	*pvdS* in frame deletion mutant, obtained by allelic exchange using pJD93 on PASK10-L	This study
PAJL176	Site-targeted Y442D mutation in *bifA,* obtained by allelic exchange using pJL108 on PAJD58	This study
PAJL186	Site-targeted D442Y mutation in *bifA*, obtained by allelic exchange using pJL103 on PAZH13-N	This study
PAJL196	Site-targeted Y442D mutation in *bifA,* obtained by allelic exchange using pJL108 on PAO1-L	This study
<u>*E. coli*</u>		
DH5α	F^- *endA1 glnV44 thi-1 recA1 relA1 gyrA96 deoR nupG purB20* φ80d*lacZ*ΔM15 Δ(*lacZYA-argF*)U169, hsdR17($r_K^- m_K^+$), λ^-	[4]
S17.1 λpir	*pro, res− hsdR17 (rK− mK+) recA−* with an integrated *RP4-2-Tc::Mu-Km::Tn7*, Tpr λpir	[5]
BL21 (DE3)	F^- *ompT gal dcm lon hsdS*$_B$($r_B^- m_B^-$) λ(DE3 [*lacI lacUV5-T7p07 ind1 sam7 nin5*]) [*malB*$^+$]$_{K\text{-}12}$(λ^S)	[6]

**Plasmids**		
pBluescript-II KS+	Cloning vector; ColE1 replicon; Amp^R	Stratagene
mini-CTX*lux*	Promoter-probe vector containing the *luxCDABE* operon; Tc^R	[7]
pME6000	pBBR1MCS-derived broad host range multicopy vector, Tc^R	[8]
pME6032	pVS1-p15A shuttle expression vector; IPTG inducible; Tc^R	[9]
pME3087	Suicide vector, ColE1 replicon, Mob; Tc^R	[10]
pDM4	Suicide vector; *sacBR*, oriR6K; Cm^R	[11]
pHP45Ω	Source of ΩSm/Spc interposon; Amp^R	[12]
pBAD TOPO TA	Topoisomerase I-activated vector for cloning of *Taq*-amplified PCR products, *araBAD* promoter for regulated expression, C-terminal polyhistidine (6xHis) tag; Amp^R	Invitrogen
pUCP18	pUC18 derivative containing a stabilising fragment for maintainance in *Pseudomonas*; Amp^R, E.coli/Cb^R, *P. aeruginosa*	[13]
pCMVDsRed-Express2	pCMV-based vector harbouring *dsRed-express2*; Amp^R	Takara Bio USA, Inc
P_{cdrA} - *gfp*(ASV)^C	pUCP22Not-P_{cdrA}-RBSII-*gfp*(ASV)-T0-T1; Amp^R, $Gent^R$	[14]
pMRE147	pPROBE-based vector harbouring mClover3; Cm^R, $Gent^R$	[15]
pAMP11	mini-CTX*lux* based transcriptional fusion containing P_{toxR1} promoter; Tc^R	This study
pAMP12	mini-CTX*lux* based transcriptional fusion containing P_{toxR2} promoter; Tc^R	This study
pAMP13	mini-CTX*lux* based transcriptional fusion containing $P_{toxR1,2}$ promoter; Tc^R	This study
pSH30	mini-CTX*lux* based transcriptional fusion containing P_{rhlA} promoter; Tc^R	This study
pSH32	mini-CTX*lux* based transcriptional fusion containing P_{rhlI} promoter; Tc^R	This study
pSC581	pBluescript II KS^+ derivative containing *dsRed-express2*; Amp^R.	This study
pSC855	pUCP18 derivative containing P_{pel} - *dsRed* transcriptional fusion; Amp^R	This study
pSC856	pUCP18 derivative containing P_{psl} - *dsRed* transcriptional fusion; Amp^R	This study
pMMB-1	pME6000 derivative harbouring a genomic fragment of 2.2 kb; Tc^R	This study
pMMB-2	pME6000 derivative harbouring a genomic fragment of 2.7 kb; Tc^R	This study

pMMB-3	pME6000 derivative harbouring a genomic fragment of 2 kb; Tc^R	This study
pMMB-4	pME6000 derivative harbouring a genomic fragment of 3 kb; Tc^R	This study
pMMB-5	pME6000 derivative harbouring a genomic fragment of 3 kb; Tc^R	This study
pMMB-6	pME6000 derivative harbouring a genomic fragment of 3.7 kb; Tc^R	This study
pMMB-7	pME6000 derivative harbouring a genomic fragment of 2.7 kb; Tc^R	This study
pMMB-8	pME6000 derivative harbouring a genomic fragment of 3.5 kb; Tc^R	This study
pMMB-10	pME6000 derivative harbouring a genomic fragment of 3.5 kb; Tc^R	This study
pMMB-11	pME6000 derivative harbouring a genomic fragment of 3.1 kb; Tc^R	This study
pMMB-13	pME6000 derivative harbouring a genomic fragment of 3.1 kb; Tc^R	This study
pMMB-14	pME6000 derivative harbouring a genomic fragment of 3.5 kb; Tc^R	This study
pMMB-16	pME6000 derivative harbouring a genomic fragment of 2.5 kb; Tc^R	This study
pJD22	pDM4 suicide plasmid with upstream and downstream regions of *toxR* joined together for the generation of *toxR* in frame deletion; Cm^R	This study
pAMP5	pDM4 suicide plasmid for the generation of the *toxR*-inducible strain; Sm/Spc-*lacIQ*-Ptac-*toxR*; Sm^R, Spc^R	This study
pJD93	pME3087 suicide plasmid containing upstream and downstream regions of *pvdS* joined together for the generation of *pvdS* in frame deletion; Tc^R	This study
pJL103	pME3087 suicide plasmid harbouring *bifA* with a Y442D point mutation from PAO1-N; Tc^R	This study
pJL108	pME3087-based suicide plasmid harbouring *bifA* from PAO1-L; Tc^R	This study
pAMP14	pBAD TOPO-based vector harbouring *toxR*-6His; Amp^R	This study
pZH13	pDM4 suicide plasmid with upstream and downstream regions of *rsmA* joined together for the generation of *rsmA* in frame deletion; Cm^R	[2]
**Oligonucleotides**	**Sequence (5'-3')[#]**	**Modification**
ToxRp1FW	TATAAGCTTGCGATATCCATTTCGTCTGG	HindIII
ToxRp1RV	TATGAATTCAGGGTACTTCCGGTGTCGC	EcoRI
ToxRp2FW	TATAAGCTTCGGCGGCCGATTGCCGA	HindIII

ToxRp2RV	TATGAATTCGCGCGTTATCGGATGATTAGG	EcoRI
ToxRp12FW	TATAAGCTTGCGATATCCATTTCGTCTGG	HindIII
ToxRp12RV	TATGAATTCGCGGAGACGACCCTACCTC	EcoRI
RhlApFW	TATCTCGAGGTTTCGACACCGGAAACCG	XhoI
RhlApRV	TATCTGCAGTACCAACAGACTTTCGCGCC	PstI
RhlIpFW	TATCTCGAGCGCTGGGTCTCATCTGAAG	XhoI
RhlIpRV	TATCTGCAGCGATTCAGAGAGCAATTCGA	PstI
P*pelAdsRed*FW	ATAGGTACCACGCAACTGAAGGCGGTGCGA	KpnI
P*pelAdsRed*RV	ATAAAGCTTCCATGCCCAGCCTACGCGGCA	HindIII
P*pslAdsRed*FW	ATAGGTACCTCTTCCGCCTTCGACGAGGGC	KpnI
P*pslAdsRed*RV	ATAAAGCTTCCATGTTGTTTGCTCTGCCGA	HindIII
*dsRed*FW	ATAGGATCCATGGaagcttTGGATAGCACT*	BamHI
*dsRed*RV	ATAGAATTCCTACTGGAACAGGTGGTGG	EcoRI
ToxRtopoFW	GAGGAATAATAAATGACTGCGACAGACAGAACG CCCC	N/A
ToxRtopoRV	GCAGGCCGGACTGCTGAACGGCCA	N/A
*rsmA*D1FW	GCTCTTGATTTCTGCGGATCCGCCGCC	BamHI
*rsmA*D1RV	GGTCTCTCCGACCCGTCTAGATTAAATCAGCAT TCC	XbaI
*rsmA*D2FW	CATCCAGAAAGAGAAAGGTACCGAGCCAAACCA TTAA	KpnI
*rsmA*D2RV	CGGTACCCTGCAGGCCCTTTCGGTATGGCGCA C	KpnI
*toxR*D1FW	TATGAATTCGCGATATCCATTTCGTCTGG	EcoRI
*toxR*D1RV	ATAGGATCCAGAGCTCGAATCCGTCGTT	BamHI
*toxR*D2FW	ATAGGATCCCACTATTCGGAGCCTTTCCA	BamHI
*toxR*D2RV	ATATCTAGAAAGACAGGCTCTTCGTGCAT	XbaI
*toxR*pFW	TATAAGCTTGCGATATCCATTTCGTCTGG	HindIII
*toxR*pRV	TATGAATTCAAGTGATGGCTCTATGGGC	EcoRI
*pvdS*D1FW	TATAAAGCTTATAGCGATGTACGCCATGGA	HindIII
*pvdS*D1RV	TATAGAATTCGTATCGCATCTGCGGGTAGA	EcoRI
*pvdS*D2FW	TATAGAATTCGGTGCACTGCCGCAAGGTCA	EcoRI
*pvdS*D2RV	TATATCTAGAACCTGCGCATTCATCGAGGA	XbaI
BifAFW	TATGAATTCAGCAACTCGACCAGATCCTC	EcoRI
BifARS	TATAAGCTTGGTCCTGGACGAAACTCTTG	HindIII

** Lowercase bases indicate an internal HindIII restriction site located 1bp from the ATG translational start site in dsRed coding nucleotide sequence.*

#nucleotide sequences of restriction sites are underlined.

SUPPLEMENTARY FIGURES

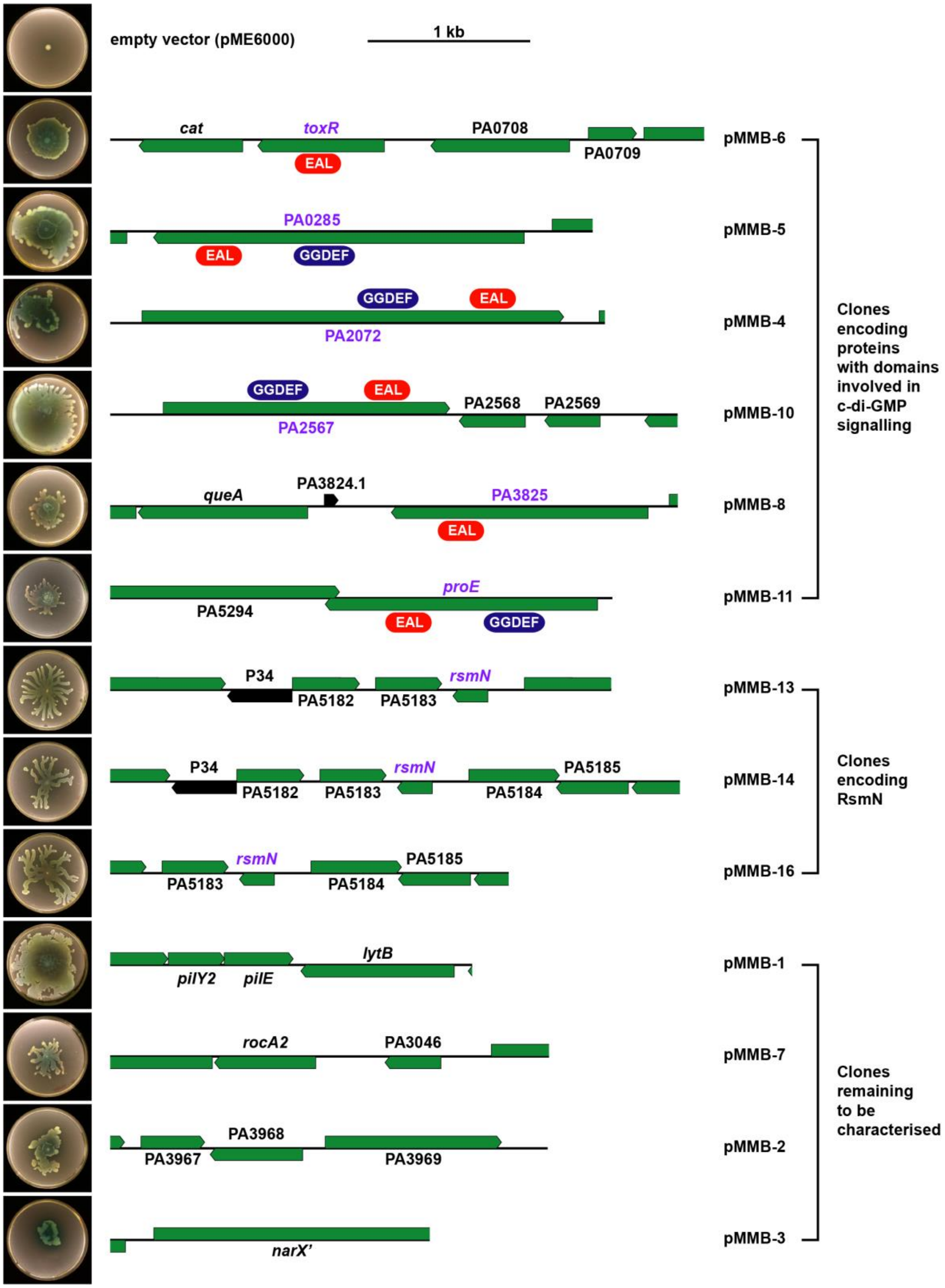

Supplementary Figure 1. Clones complementing swarming motility in PAO1-N. DNA fragments from PAO1-L WT restoring swarming motility (insert images) in the PAO1-N *ΔrsmA* mutant when overexpressed from the pME6000 vector. Typical EAL (red framed) and GGDEF (blue framed) domains of proteins involved in c-di-GMP metabolism were identified in the predicted proteins. Green arrows represent ORFs and black arrows non-coding RNAs. Genes labelled purple were identified as complementing swarming in PAO1-N *ΔrsmA* mutant when individually sub-cloned.

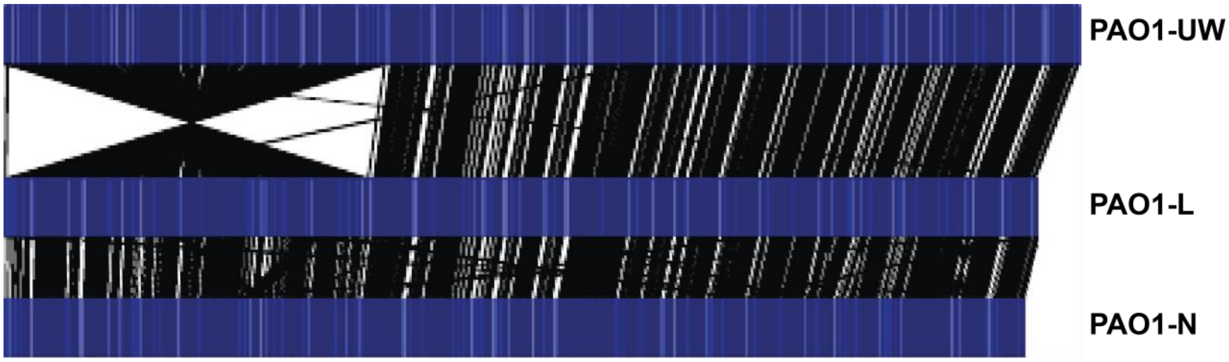

Supplementary Figure 2. The 2.2-Mb inversion is not present in the PAO1 sublines used in this study. Alignment of the optical map of PAO1-UW reference sequence (Genbank Accession Number: NC 002516) produced *in silico* with the optical maps of PAO1 sublines PAO1-L or PAO1-N obtained experimentally. Optical maps are shown in blue, and the alignment of restriction enzyme cut sites between optical maps are shown in black.

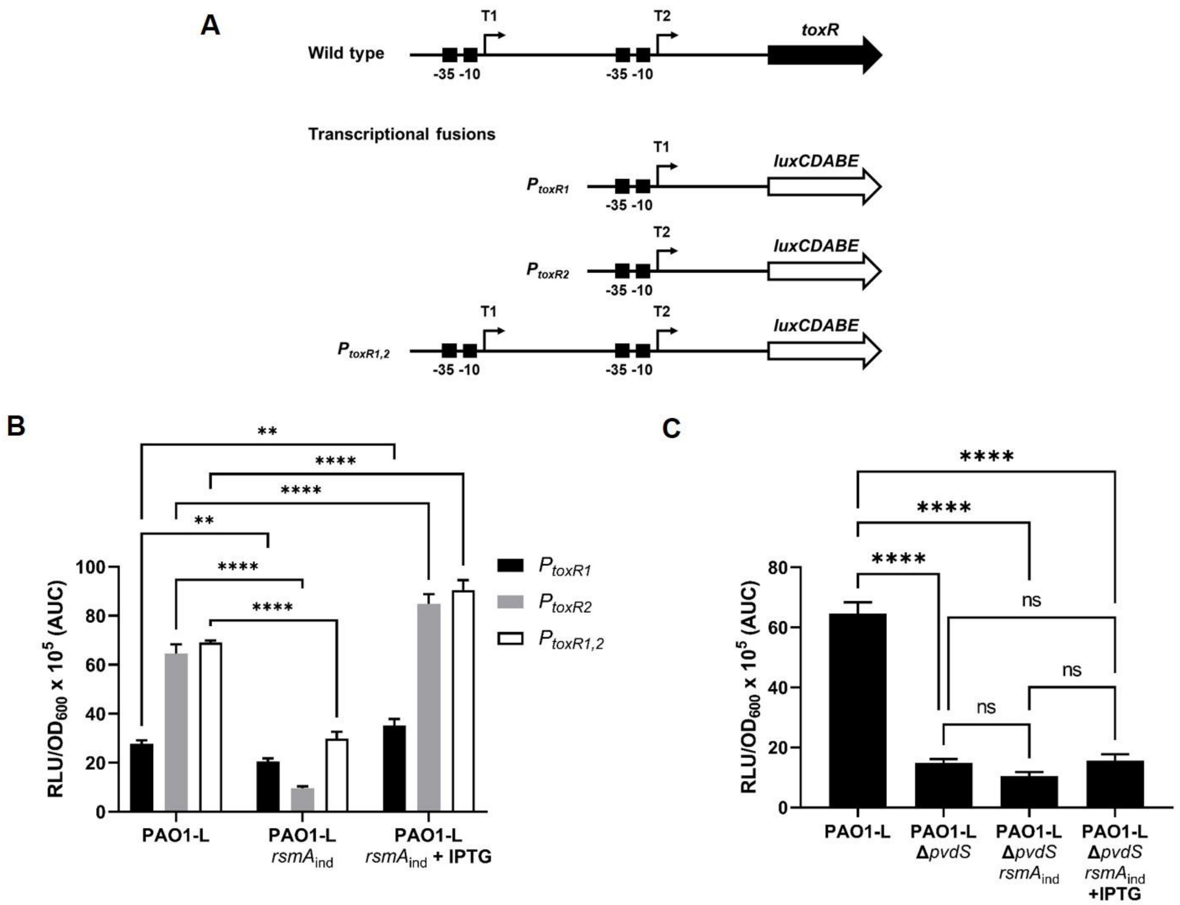

Supplementary Figure 3. Effect of RsmA and PvdS on *toxR* expression. (A) Schematic of the *toxR* promoter region used to build mini-CTX::P_{toxR1}, P_{toxR2} and $P_{toxR1,2}$ - *luxCDABE* transcriptional fusions. (B) Effect of *rsmA* mutation on the transcriptional activity of the *toxR* promoters measured in PAO1-L and the IPTG-inducible conditional *rsmA* mutant of PAO1-L ($rsmA_{ind}$) under ion limiting conditions in CAA medium. (C) Transcriptional activity of mini-CTX::P_{toxR2} - *luxCDABE* in *P. aeruginosa* PAO1-L wild type and its corresponding mutants Δ*pvdS* and Δ*pvdS* $rsmA_{ind}$ measured under iron starvation in CAA medium. Cells were grown in a 96-well microplate. Bioluminescence and absorbance (OD_{600}) were measured using an automated luminometer-spectrometer (TECAN Genios Pro). Reported values are averages from three different cultures ±

standard deviation and correspond to the area under the curve (AUC) derived from plotting relative luminescence units normalized to culture density (RLU/OD_{600}) over time (24 h). Statistical differences between group means were determined by two-way ANOVA tests. (*$p < 0.05$, **$p < 0.01$, ***$p < 0.005$, ****$p < 0.0001$).

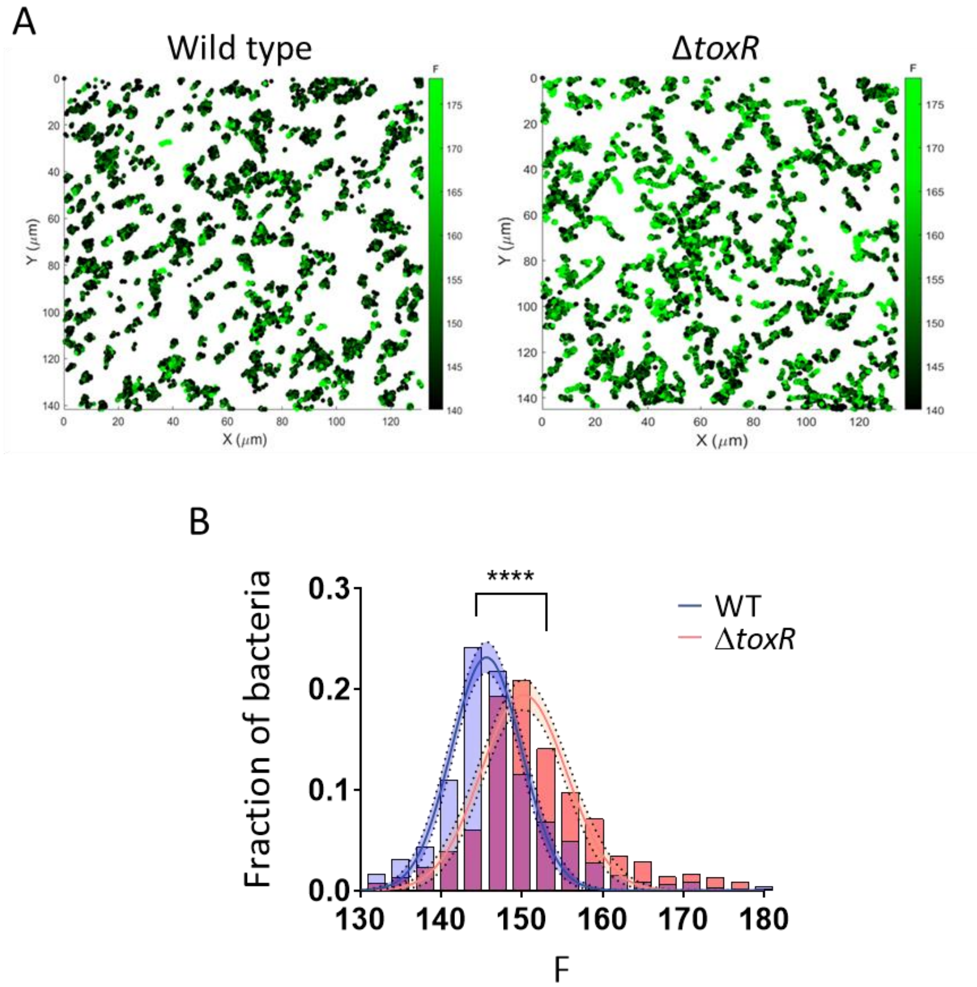

Supplementary Figure 4. Impact of ToxR on the levels of *cdrA* expression at individual cell level within the bacterial population. (A) Representative coloured maps of PAO1-L WT and PAO1-L Δ*toxR* mutant cells expressing the P_{cdrA} - *gfp*(ASV)^{C} transcriptional fusion reporter. Colours show instantaneous fluorescence intensity measured (F) according to the intensity scale shown. Tracks were obtained from combined images acquired using DIC and widefield epifluorescence microscopy every 30 s for 60 min and then processed through a custom single-cell tracking algorithm. (B) Histogram of F for all tracks on PAO1-L WT and Δ*toxR* mutant (N > 1,000). Unpaired t-

test with Welch's correction was used to compare curves (****p < 0.0001). Dashed lines represent data fit to Gaussian functions. Shaded areas represent 95% CI of the fits.

```
             Sid     1                                                                              80
Cd01948 100%    ADLRRALERGEFELYYQPIVDLRTGRIVGY--EALLRWRHPEGGL-ISPAEFIPLAEETGLIVELGRWVLEEACRQLARW
PA2133  27.2%   RRFQAALARGRVRLDWQAVRHAGHPEWEPLYRETLLRVTAASGEPPLPTQELILALERLGLVRLLDRCVLGTVLDRLN--
PA2200  36.4%   ARLLRALKREALEVHYQPIVRLASGRCAGV--EALARWTDSSLGR-VSPDVFIGALEESGDIELLTRFVFRRALKQLGPL
PA2818  39.2%   GDMYRAMRAREFHMVYQPIIHLDTGECRGV--EALVRWQRPDRSQ-VRPDIFIPLAEDNGMIGDLTRHIFGLVAADLAQL
PA3825  41.7%   SELRRALEANEFIPYYQPLSPGQGGRWIGV--EVLMRWRHPREGL-IRPDLFIPFAERSGLIVPMTRALMRQVAEDLGGH
RocR    38.6%   ADVVRGLDNGEFEAYYQPKVALDGGGLIGA--EVLARWNHPHLGV-LPPSHFLYVMETYNLVDKLFWQLFSQGLATRRKL
FimX    33.5%   AILQQALETNSFRLLFQPVISLRGDSHENY--EVLLRLLNPQGQE-VPPAEFLHAAKEAGLAEKIDRWVILNSIKLLAEH
LapD    30.5%   DWIDQALTERRLLLYFQPVVDCLDTQRV-LHHKVLARLLDPQATA-IAAGRFLPWIERFGWAARMDLAMLEQSLEHLRR-
ToxR    20.0%   WLCLGNRDANDGFELFAHGIYARNGALVGS--KLSLRERRQRVDLSAFLSGAPPLLAEAAVKHLLARLLCVHRHNT----

                 81                                                                             160
Cd01948 100%    Q-AGGPDLRLSVNLSARQLRDPDFLDRLLELLAETGLPPRRLVLEITESALIDDLEEALATLRRLRALGVRIALDDFGTG
PA2133  27.2%   ---AEPTLRLACNLSRQSAAMDAWWEAVCRWLAARPQVARRLTLELTETAVGERVA-TREFIRRLREHGVRIAIDDFGAA
PA2200  36.4%   L-REQRSFYVSVNVTGKDIADPGFIDFAMRQMARESVRPEQVALELTERTT-EAQGCLLAGMNRLRELGLKIYVDDFGTG
PA2818  39.2%   G--LGAGDHLGVNVSGSHLASHGFVDDVRRLLGAIGSEGPQLVLEVTEREALPHDAQLQHNIQQLRELGVQWALDDFGTG
PA3825  41.7%   AGKLEPGFHIGFNISATHCHELALVDDCRELLAAFPPGHITLVLELTERELIESSEVTDRLFDELHALGVKIAIDDFGTG
RocR    38.6%   A-QLGQPINLAFNVHPSQLGSRALAENISALLTEFHLPPSSVMFEITETGLISAPASSLENLVRLRIMGCGLAMDDFGAG
FimX    33.5%   R-AKGHQTKLFVHLSSASLQDPGLLPWLGVALKAARLPPESLVFQISEADATSYLKQAKQLTQGLATLHCQAAISQFGCS
LapD    30.5%   -----HPRPLALSLSAASVRNAQTFAPLLALLKAHPQEARQLTLELDERHL-PAAAELERLSQVLRELGCGLGLQHFGGR
ToxR    20.0%   --DLELLGKNFIPLHASSLGNAGVCERILASARQLQQHQVELCLLLAIDEQEPASAEYLTSLARLRDSGVRIALHPQRID

                 161                                                                            240
Cd01948 100%    YSSLSYLKRLPV---DYLKIDRSFVRD--IETDPEDRAIVRAIIALAHSLGLKVVAEGVETEEQLELLRELGCD-YVQGY
PA2133  27.2%   HNNLDFVLDARP---DVIKIDCRYTRE--ARRSAKGAEVLRHLLALCRELAPCVVLEGLEEDDAFARLPTGDV--YLQGN
PA2200  36.4%   HSNLVYLANLPV---DAIKIDKVFTQS--IGDSSAVELIFDKLCSMAEHLEIGVVVEGIETQAQADHVLRRSPEALGQGW
PA2818  39.2%   QSSLSHLQKLHA---DFLKIDRSFVSS--VGSGSVNAVVLETIIALAQRLDLAMTAEGIETREQEQYLCGHSIQ-WGQGY
PA3825  41.7%   HSSLAYLRKFQV---DCLKIDQSFVAR--IGIDTLSGHILDSIVELSAKLDLDIVAEGVETPEQRDYLAARGVD-YLQGY
RocR    38.6%   YSSLDRLCEFPF---SQIKLDRTFVQK--MKTQPRSCAVISSVVALAQALGISLVVEGVESDEQRVRLIELGCS-IAQGY
FimX    33.5%   LNPFNALKHLTV---QFIKIDGSFVQD--LNQVENQEILKGLIAELHEQQKLSIV-PFVESASVLATLWQAGAT-YIQGY
LapD    30.5%   FSLIGNLTHLGL---AYLKLDGCYLHA--VDREGDKRLFIEAVYRTTHSIDLPLIAEQVETLGELEVLREMGLR-GAMGR
ToxR    20.0%   TDARQCFAEVDAGLCDYLGLDARLLAPGPLTRNLRQRKSIEYLNRLLVAQDIQMLCLNVDNEELHQQANALPFA-FRHGR
```

Supplementary Figure 5. Level of conservation between the ToxR EAL domain and other c-di-GMP binding proteins in *P. aeruginosa*. Clustal Omega Alignment of the conserved active EAL site (cd01948) and EAL sequences from EAL-only proteins of *Pseudomonas aeruginosa* PAO1 including ToxR. The residues that form the active site of c-di-GMP PDEs are highlighted in red; conserved residues located in the vicinity of the active site are highlighted in green.

SUPPLEMENTARY REFERENCES

1. Stover, C. K. *et al.* Complete genome sequence of *Pseudomonas aeruginosa* PAO1, an opportunistic pathogen. *Nature* **406**, 959-964, doi:10.1038/35023079 (2000).
2. Pessi, G. *et al.* The global posttranscriptional regulator RsmA modulates production of virulence determinants and N-acylhomoserine lactones in *Pseudomonas aeruginosa*. *Journal of bacteriology* **183**, 6676-6683, doi:10.1128/jb.183.22.6676-6683.2001 (2001).
3. Kulkarni, P. R. *et al.* A sequence-based approach for prediction of CsrA/RsmA targets in bacteria with experimental validation in *Pseudomonas aeruginosa*. *Nucleic acids res* **42**, 6811-6825, doi:10.1093/nar/gku309 (2014).
4. Hanahan, D. Studies on transformation of *Escherichia coli* with plasmids. *J Mol Biol* **166**, 557-580, doi:10.1016/s0022-2836(83)80284-8 (1983).
5. Metcalf, W. W., Jiang, W. & Wanner, B. L. Use of the rep technique for allele replacement to construct new *Escherichia coli* hosts for maintenance of R6K gamma origin plasmids at different copy numbers. *Gene* **138**, 1-7, doi:10.1016/0378-1119(94)90776-5 (1994).
6. Studier, F. W. & Moffatt, B. A. Use of bacteriophage T7 RNA polymerase to direct selective high-level expression of cloned genes. *J Mol Biol* **189**, 113-130, doi:https://doi.org/10.1016/0022-2836(86)90385-2 (1986).
7. Becher, A. & Schweizer, H. P. Integration-proficient *Pseudomonas aeruginosa* vectors for isolation of single-copy chromosomal *lacZ* and *lux* gene fusions. *Biotechniques* **29**, 948-950, 952 (2000).
8. Maurhofer, M. *et al.* Salicylic Acid Biosynthetic Genes Expressed in *Pseudomonas fluorescens* Strain P3 Improve the Induction of Systemic Resistance in Tobacco Against Tobacco Necrosis Virus. *Phytopathology* **88**, 678-684, doi:10.1094/PHYTO.1998.88.7.678 (1998).
9. Heeb, S., Blumer, C. & Haas, D. Regulatory RNA as mediator in GacA/RsmA-dependent global control of exoproduct formation in *Pseudomonas fluorescens* CHA0. *Journal of bacteriology* **184**, 1046-1056, doi:doi:10.1128/jb.184.4.1046-1056.2002 (2002).
10. Voisard, C. *et al.* in *Molecular ecology of rhizosphere microorganisms* 67-89 (1994).
11. Milton, D. L., O'Toole, R., Horstedt, P. & Wolf-Watz, H. Flagellin A is essential for the virulence of *Vibrio anguillarum*. *J Bacteriol* **178**, 1310-1319 (1996).
12. Prentki, P. & Krisch, H. M. In vitro insertional mutagenesis with a selectable DNA fragment. *Gene* **29**, 303-313 (1984).
13. Schweizer, H. P. *Escherichia-Pseudomonas* shuttle vectors derived from pUC18/19. *Gene* **97**, 109-121, doi:10.1016/0378-1119(91)90016-5 (1991).
14. Rybtke, M. T. *et al.* Fluorescence-based reporter for gauging cyclic di-GMP levels in *Pseudomonas aeruginosa*. *Appl Environ Microbiol* **78**, 5060-5069, doi:10.1128/aem.00414-12 (2012).
15. Schlechter, R. O. *et al.* Chromatic bacteria – a broad host-range plasmid and chromosomal insertion toolbox for fluorescent protein expression in bacteria. *Front Microbiol* **9**, doi:10.3389/fmicb.2018.03052 (2018).
